# Neurotrophic factor-α1, a novel tropin is critical for the prevention of stress-induced hippocampal CA3 cell death and cognitive dysfunction in mice: comparison to BDNF

**DOI:** 10.1038/s41398-020-01112-w

**Published:** 2021-01-07

**Authors:** Lan Xiao, Vinay Kumar Sharma, Leila Toulabi, Xuyu Yang, Cheol Lee, Daniel Abebe, Areg Peltekian, Irina Arnaoutova, Hong Lou, Y. Peng Loh

**Affiliations:** Section on Cellular Neurobiology, Eunice Kennedy Shriver National Institute of Child Health and Human Development, National Institutes of Health, Bethesda, MD 20892 USA

**Keywords:** Molecular neuroscience, Hippocampus

## Abstract

Stress leads to brain pathology including hippocampal degeneration, cognitive dysfunction, and potential mood disorders. Hippocampal CA3, a most stress-vulnerable region, consists of pyramidal neurons that regulate cognitive functions e.g. learning and memory. These CA3 neurons express high levels of the neuroprotective protein, neurotrophic factor-α1 (NF-α1), also known as carboxypeptidase E (CPE), and receive contacts from granule cell projections that release BDNF which has neuroprotective activity. Whether NF-α1-CPE and/or BDNF are critical in protecting these CA3 neurons against severe stress-induced cell death is unknown. Here we show that social combined with the physical stress of maternal separation, ear tagging, and tail snipping at weaning in 3-week-old mice lacking NF-α1-CPE, led to complete hippocampal CA3 degeneration, despite having BDNF and active phosphorylated TrkB receptor levels similar to WT animals. Mice administered TrkB inhibitor, ANA12 which blocked TrkB phosphorylation showed no degeneration of the CA3 neurons after the weaning stress paradigm. Furthermore, transgenic knock-in mice expressing CPE-E342Q, an enzymatically inactive form, replacing NF-α1-CPE, showed no CA3 degeneration and exhibited normal learning and memory after the weaning stress, unlike NF-α1-CPE-KO mice. Mechanistically, we showed that radio-labeled NF-α1-CPE bound HT22 hippocampal cells in a saturable manner and with high affinity (Kd = 4.37 nM). Subsequently, treatment of the HT22^*cpe−/−*^ cells with NF-α1-CPE or CPE-E342Q equivalently activated ERK signaling and increased BCL2 expression to protect these neurons against H_2_O_2_-or glutamate-induced cytotoxicity. Our findings show that NF-α1-CPE is more critical compared to BDNF in protecting CA3 pyramidal neurons against stress-induced cell death and cognitive dysfunction, independent of its enzymatic activity.

## Introduction

Hippocampal degeneration, impaired neuronal network, reduced neurogenesis, and cognitive dysfunction are pathological changes that occur after stress^1,2^ and in neurodegenerative disorders such as Alzheimer’s disease (AD)^2,3^. Dysfunction of a variety of neurotrophic factors such as NGF, BDNF, NT3, and GDNF, have been shown to be involved in the pathogenesis of neurodegeneration. Each neurotrophic factor is highly expressed and involved in the survival, proliferation, and neuroprotection of certain types of neurons and is significantly decreased in specific areas of degeneration in post-mortem brains of AD patients^4–7^. However, the currently known neurotrophic factors, including BDNF do not appear to adequately account for the neuroprotective effects of the highly stress-vulnerable CA3 pyramidal neurons of the hippocampus observed during stress, such as during global ischemia in rats^8^. Early studies showed that carboxypeptidase E (CPE) mRNA which encodes a prohormone/proneuropeptide processing enzyme^9,10^ and the protein, are highly expressed in hippocampal CA3 neurons, and are significantly increased in a sustained manner after global ischemia, facilitating the survival of these neurons in rats^8^. In contrast, CA1 neurons which showed only a transient rise in CPE were vulnerable to ischemia. Likewise, after chronic restraint stress, CPE mRNA was significantly up-regulated in the CA1-CA3 region of the hippocampus in mice. Moreover, CPE-knockout (KO, *cpe*^−/−^) mice after a weaning stress paradigm at 3 weeks of age, which included maternal separation, ear tagging, and tail snipping, exhibited complete hippocampal CA3 degeneration by week 4, as well as diminished neurogenesis in the dentate gyrus^11,12^. However, CPE-KO mice that were not subjected to this weaning stress at 3 weeks of age showed an intact CA3 region in the hippocampus when observed at week 4, indicating that the degeneration is linked to the stress^13^. These abnormalities were accompanied by inability to elicit long-term potentiation with tetanic stimulation of the hippocampus and profound deficits in learning and memory function as evidenced by poor performance in the Morris water maze test, social transmission of food preference, and object preference tests^11^. When NF***-***α1-CPE-KO animals did not undergo weaning at 3 weeks of age, there was no degeneration of the hippocampal CA3 region, indicating that it is not an intrinsic developmental defect, but rather one uncovered upon environmental stress challenge after postnatal development. Furthermore, when the NF***-***α1-CPE-KO mice were treated with the anti-epileptic drug, carbamazepine, prior to weaning, there was no degeneration of the hippocampal CA3 region following the weaning stress paradigm^13^. This finding suggests that the degeneration of the CA3 neurons in this case was caused by glutamate excitotoxicity. Since BDNF-containing granule cell mossy fiber boutons contact and release BDNF onto the CA3 pyramidal neurons, and the BDNF immunoreactivity increased in the mossy fibers after mice underwent status epilepticus^14^, this neurotrophin is thought to be involved in protecting these CA3 neurons from stress^15,16^. Based on the studies of McEwen’s group^1^, we propose that emotional and physical stress incurred during the weaning paradigm, or restraint stress, which causes a huge increase in glucocorticoid release (Supplementary Fig. S1), would stimulate the granule cells in the dentate gyrus to release glutamate via the mossy fibers that contact the pyramidal neurons in the CA3 region. This then results in excitotoxicity and complete degeneration of these neurons observed in NF***-***α1-CPE-KO mice, leading to cognitive dysfunction.

In the present study, we addressed two questions: (1) What is the contribution of NF***-***α1-CPE versus BDNF in preventing degeneration of the CA3 neurons and cognitive decline in mice with severe excitotoxic stress? (2) Since NF***-***α1-CPE is required to enzymatically process certain neuropeptides that may be involved in neuroprotection^17^, is it acting extracellularly as a neuroprotective factor independent of its enzymatic activity, or playing a role by generating active neuropeptides needed to neuroprotect CA3 neurons in vivo? To answer the first question, we have examined the levels of BDNF and phosphorylated TrkB in the hippocampus of NF-α1-CPE-KO mice before weaning and after the weaning stress paradigm. We also determined the effect of inhibiting the activation of TrkB, the BDNF receptor with ANA12 injected into WT mice at 2 weeks of age for 14 days and then examining the CA3 region for neurodegeneration after the weaning stress paradigm. To address the second question, we delineated the enzymatic from the neurotrophic functions of NF-α1-CPE, by constructing a mutant form of mouse NF-α1-CPE that lacks the enzyme activity by substituting glutamate (E) at position 342 to glutamine (Q), based on rat studies which showed a mutation of CPE, E300Q eliminated enzyme activity^18^. We then generated knock-in mice, replacing WT-CPE with enzymatically inactive CPE-E342Q, and determined if these mice were protected from stress-induced CA3 degeneration and cognitive decline. We also investigated the effect of recombinant CPE-E342Q on neuroprotection of HT22^*cpe−/−*^ hippocampal cell line and primary hippocampal neurons under cytotoxic stress, and the receptor-mediated signal transduction mechanism involved. Our studies show that NF***-***α1-CPE, acting independent of its enzymatic activity, surpasses BDNF as a critical factor in protecting hippocampal CA3 neurons from severe stress-induced degeneration through interacting with a high-affinity receptor which activated the ERK-BCL2 signaling pathway.

## Material and methods

### Animals

All animals were housed at NIH animal facility with free access to food and water ad libitum and controlled humidity (45%) and temperature (22 °C) under a 12 h light/dark cycle. A knock-in CPE-E342Q mouse model expressing enzymatically inactive CPE was generated as described in Supplementary Methods. The genetic background of CPE-E342Q and CPE-KO are C57BL/6J and homozygous mice were produced by mating male and female heterozygote mice due to infertility of homozygotes with WT littermates as control. Homozygous offsprings resulting from breeding heterozygotic parents are born with lower than expected frequency and therefore difficult to obtain large numbers of homozygous animals. Mice were weaned, ear-tagged, and tails clipped for genotyping at 3 weeks of age. All animal study protocols complied with ethical regulations and were approved by the Animal Care and Use Committee of NICHD, NIH. These mouse models could be made available upon request.

### Weaning, toe clipping, and ANA12 injection of mice

To verify no degeneration in the hippocampus in all 3 genotypes (WT, CPE-E342Q, and CPE-KO) after toe clipping procedure within postnatal day 7, newborns were toe-clipped for tagging and subsequently genotyped within 7 days after birth, and sacrificed at week 3 without weaning for evaluation by Nissl staining (Supplementary Fig. S2). To study the effect of ANA12, a TrkB inhibitor, on neuroprotection, mice were toe clipped within postnatal day 7 for genotyping and randomly received either vehicle or ANA12 (Sigma-Aldrich, St. Louis, MO, Cat. #SML0209) (0.5 mg/kg) injection i.p. for 14 days starting from week 2 to week 4, with weaning stress at week 3, and then sacrificed at week 4 for Nissl staining or biochemical studies.

### Organotypic hippocampal slice culture

Hippocampal slices were prepared according to the membrane interface method^19^. Hippocampi were dissected from 5- to 6-day-old WT and CPE-KO mouse brains. Isolated hippocampi were cut into slices with 300 μm thickness using a tissue chopper (McIlwain) and transferred to 0.4 μm cell culture inserts (Millipore, Burlington, MA) that were placed in six-well culture plates. Slices were cultured in the medium with 50% MEM, 25% HBSS, 25% heat-inactivated horse serum (Life Technologies, Carlsbad, CA), 0.64% glucose (Sigma, St. Louis, MO), 0.04% NaHCO3. Slices were incubated at 35 °C, 5% CO_2_, humidified condition and the culture medium was changed the first day after preparation then every second day. To induce excitotoxicity, slices were treated by 5 μM kainic acid (Sigma, St. Louis, MO) for 18 h and dead cells were detected by propidium iodide (Life Technologies, Carlsbad, CA, 1 μg/mL) staining.

### Cell lines and generation of the HT22^*cpe−/−*^ cells

HT22, a mouse hippocampal cell line obtained from Salk Institute (La Jolla, CA) were cultured in DMEM (Millipore Sigma, Billerica, MA), supplemented with 10% fetal bovine serum (FBS), 100 U/ml penicillin, 100 μg/ml streptomycin, 0.25 μg/ml fungizone, and 25 mM 4-(2-hydroxyethyl)-1-piperazineethanesulfonic acid (HEPES), (Thermo Fisher Scientific, Waltham, MA) at 37 °C in 5% CO_2_. CPE knockout HT22 cells (HT22^*cpe−/−*^**)** were generated by CRISPR-Cas9 method as described in Supplementary Methods.

All the cells in the experiments were monitored by light microscopy for possible mycoplasma contamination and used only if they looked healthy.

### Assay for CPE activity

Enzymatic activity of recombinant WT-CPE or CPE-E342Q (GenScript, Piscataway, NJ) and mouse brain extracts were tested using dansyl-Phe-Ala-Arg as substrate (Cambridge, ON, Canada) as previously described^20,21^ (Supplementary Methods).

### ^125^I CPE radio-ligand binding to HT22 cells

Competitive and specific binding studies were performed using ^125^I CPE and HT22 cells as described in Supplementary Methods.

### Analysis of ERK phosphorylation and BCL2 in HT22^*cpe−/−*^ cells

The effect of WT-CPE and CPE-E342Q on ERK1/2 phosphorylation and BCL2 after H_2_O_2_ challenge in HT22^*cpe−/−*^ cells were performed as described in Supplementary Methods.

### Treatment of primary mouse hippocampal neurons with CPE-WT or CPE-E342Q with and without H_2_O_2_ and other inhibitors

Embryonic hippocampal neurons were harvested from litters of embryonic day 13.5 mouse pups as previously described^22^. The cell culture and study of the neuroprotective effect of CPE-WT or CPE-E342Q in the presence of inhibitors were performed as described in Supplementary Methods.

### Behavioral studies

To evaluate the behavior of CPE-E342Q, WT, and CPE-KO mice, 8–10-week-old male and female animals were subjected to a series of behavioral studies, in the order of open field, elevated plus maze, Morris water maze, forced-swim test, sucrose preference, and restraint stress tests as described in Supplementary Methods.

The sample size was determined based on our previous studies^12,23^. All animals were randomly selected for experiments at certain ages in this study. The experimenter was blinded to the group allocation during animal experiments and the experiments were performed in a blinded manner.

### Western blot

Cell lysates and mouse brain tissues were prepared as previously described^12^. Bands were analyzed by standard western blotting procedures and visualized and quantified by the Odyssey infrared imaging system and software (LI-COR Inc, Lincoln, NE) or image J software. The protein expression level for each sample was normalized to *β*-actin or Gapdh. Antibodies are listed in Supplementary Methods.

### Immunohistochemistry and Nissl stain of mouse brains

Mice were perfused with 4% paraformaldehyde (Electron Microscopy Sciences, Hatfield, PA) and prepared as previously described^23^. The brains were sectioned coronally at 30 µm for Nissl, CPE, doublecortin (DCX), and MAP2 staining (Supplementary Methods).

### Statistical analysis

Data are representative of at least three separate experiments (N), unless specified otherwise in text. Each experiment was done in triplicates (*n* = 3). For experiments with 2 groups, data were analyzed by 2-tail Student’s *t* test, or for experiments with more than 2 groups, 1-way ANOVA, or 2-way ANOVA or 2-way repeated ANOVA followed by Tukey’s post hoc multiple comparisons tests. The variance of the data between groups was similar and indicated by the standard error of the mean (SEM) bar in the graph. Where the data showed more variance indicated by the SEM, non-parametric tests were used. Wilcoxon non-parametric comparison methods were used for analyzing the effect of kainic acid (KA) on mouse organotypic hippocampal slice culture. NPY was analyzed by Mann Whitney’s non-parametric test. Analysis was performed with the assistance of GraphPad Prism (GraphPad, La Jolla, CA) software package. Significance was set at *p* < 0.05.

## Results

### CPE-KO mice exhibit CA3 neurodegeneration after weaning stress or kainic acid treatment despite having normal BDNF expression

**Fig. 1:**
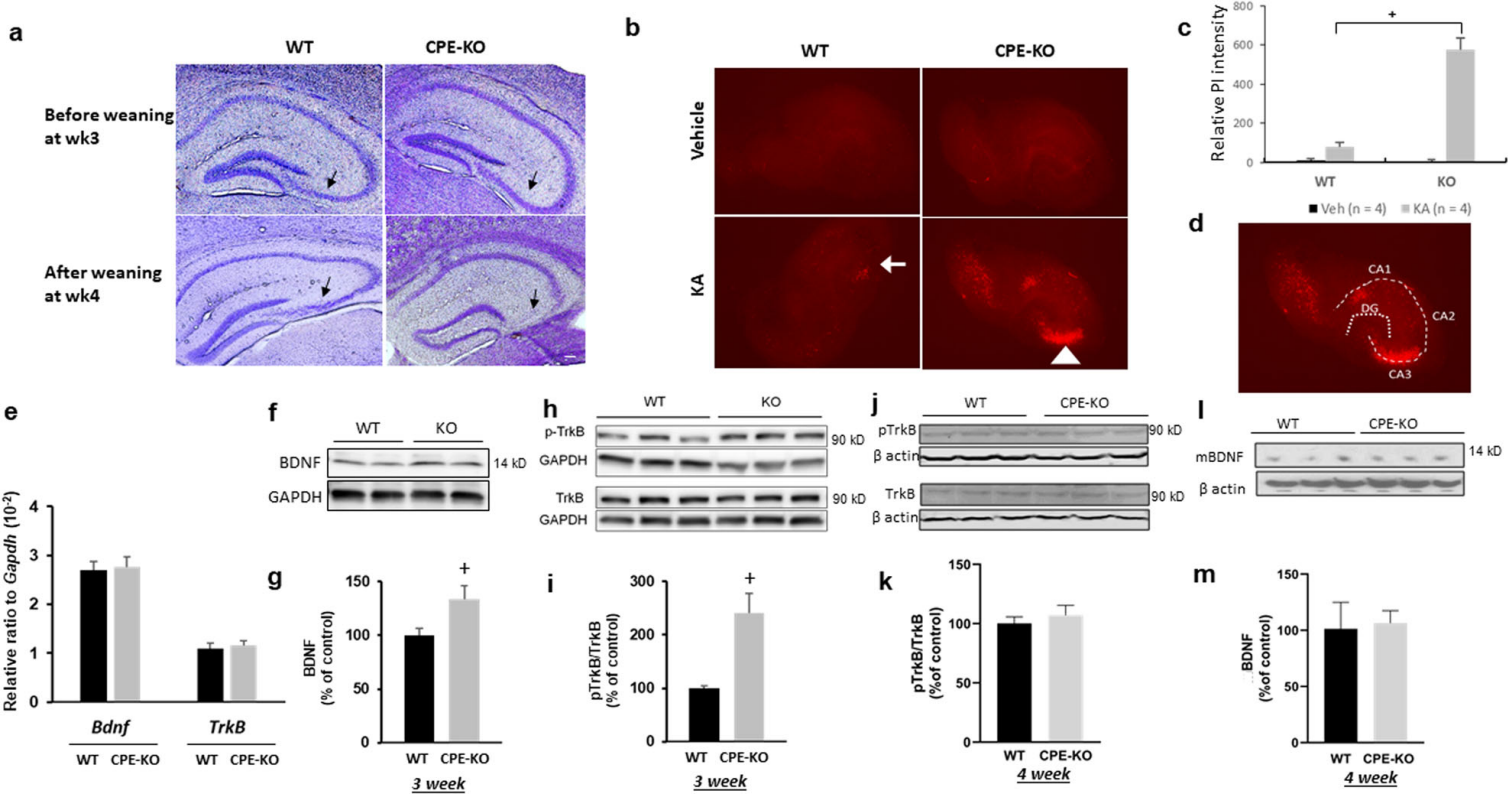
The effect of weaning stress and kainic acid on neurodegeneration of hippocampal CA3 region and BDNF/TrkB expression in hippocampus of WT and CPE-KO mice. (**a**) Nissl staining of coronal anterior hippocampus of WT and CPE-KO mice. CPE-KO mice displayed significant hippocampal CA3 degeneration after the weaning stress paradigm, but not before weaning. Sale bar = 100 μm. **b** Images of propidium iodide staining and (**c**) bar graphs representing the effect of kainic acid (KA) on mouse organotypic hippocampal slice culture. Note the severe cell death in the CA3 region (see arrowhead in (**b**)) in the CPE-KO mice compared to WT mice (see arrow). *n* = 4, Wilcoxon non-parametric comparison. ^+^*p* = 0.02857 for KA-treated KO compared with KA-treated WT. Veh: Vehicle. KA: Kainic acid. WT: wild type. **d** Schematic illustration of CA1, CA2, CA3, and dentate gyrus(DG) of the hippocampus. **e** mRNA levels of *BDNF* and *TrkB* in the hippocampus from 3-week-old CPE-KO mice. There was no difference in expression between CPE-KO and WT mice. WT *n* = 6, CPE-KO *n* = 8. The mRNA expression level for each sample was normalized to *Gapdh*. **f** Representative western blot and (**g**) quantification showing levels of BDNF protein in the hippocampus of 3-week-old CPE-KO and WT mice. *n* = 10, Student’s *t* test, ^+^*p* = 0.03314. Values are mean ± SEM. **h** Representative western blot and (**i**) quantification showing levels of phospho-TrkB and TrkB proteins in the hippocampus of 3-week-old CPE-KO and WT mice. *n* = 5. Student’s *t* test,^+^*p* = 0.01669, Values are mean ± SEM. **j** Representative Western blot and (**k**) quantification showing levels of phospho-TrkB and TrkB proteins in the hippocampus of 4-week-old CPE-KO and WT mice. *n* = 3 mice. Student’s *t* test, *p* = 0.531, values are mean ± SEM. **l** Representative Western blot and (**m**) quantification showing levels of mBDNF protein in the hippocampus of 4-week-old CPE-KO and WT mice. *n* = 3 mice, Student’s *t* test, *p* = 0.853, values are mean ± SEM.

Three-week-old WT and CPE-KO mice were subjected to the weaning stress paradigm which increased circulating cortisosterone levels (Supplementary Fig. S1), and their brains were examined. Figure 1a shows that hippocampal CA3 region from WT and the CPE-KO mice were intact before weaning at week 3. In contrast, after weaning stress, the CPE-KO mice showed complete degeneration of the CA3 region, whereas the WT mice showed an intact hippocampus, consistent with our previous findings^11^. In organotypic hippocampal slice cultures from 3-week-old WT and CPE-KO mice, treatment with 5 μM kainic for 18 h to induce excitotoxicity followed by propidium iodide staining to detect dead cells revealed that CPE-KO mice showed significant cell death in the CA3 region, but not the WT mice (Fig. 1b–d). This result suggests that the lack of CPE in the CPE-KO mice rendered the CA3 neurons more susceptible to neuronal cell death with kainic-acid-induced excitotoxicity than the WT mice. We then examined hippocampal BDNF and TrkB levels in these mice. Analysis of BDNF and TrkB mRNA in the hippocampus of 3-week-old animals showed similar levels of expression in WT and CPE-KO mice (Fig. 1e). Analysis by Western blot and quantification of BDNF protein in the hippocampus of 3-week- (Figs. 1f, g) and 4-week-old (Fig. 1l, m) mice showed higher, or equal amounts, respectively, in the CPE- KO versus WT mice. pTrkB protein was also higher at 3 weeks (Fig. 1h, i) and similar at 4 weeks (Fig. j, k) in the hippocampus of CPE-KO versus WT mice. Other growth factor levels, NGF, GDNF, and NT3 in the hippocampus were similar between CPE-KO and WT mice (Supplementary Fig. S3). Thus, despite CPE-KO mice having increased or similar levels of BDNF and pTrkB at week 3 and 4, as in WT mice, respectively, they showed complete degeneration of hippocampal CA3 region, while the WT mice had an intact CA3 region after the weaning stress paradigm (Fig. 1a). This result suggests that CPE might be more critical than BDNF in preventing CA3 neurons from cell death during stress.

### CPE-E342Q protects HT22^*cpe−/−*^ neurons from H_2_O_2_ or glutamate-induced neurotoxicity, in spite of its lack of enzymatic activity

To delineate between the enzymatic and the non-enzymatic neurotrophic function of CPE, we constructed a mutant form of CPE lacking enzymatic activity by substituting glutamate (E) 342 to glutamine (Q), and we refer to it as CPE-E342Q (Supplementary Fig. S4A). First, we demonstrated that recombinant CPE-E342Q lacks enzymatic activity using dansyl-Phe-Ala-Arg as substrate. CPE-E242Q had virtually no detectable enzyme activity (Supplementary Fig. S5A). We then investigated whether CPE-E342Q is properly trafficked, packaged into regulated secretory pathway vesicles, and secreted in an activity-dependent manner. A plasmid carrying CPE-E342Q was transfected into HT22^*cpe−/−*^ cells, a mouse hippocampal cell line engineered to eliminate endogenous CPE expression, (Supplementary Fig. S6A, B) show that expression, basal and high K^+^ stimulated (50 mM) secretion of CPE-E342Q are similar to CPE in these transfected HT22^*cpe−/−*^ cells. Transfection of CPE or CPE-E342Q prevented H_2_O_2−_ induced cytotoxicity in HT22^*cpe−/−*^ cells (Supplementary Fig. S6C). Pre-incubation of HT22^*cpe−/−*^ cells with recombinant CPE and CPE-E342Q at 25 and 50 nM effectively prevented H_2_O_2_-induced cytotoxicity (Supplementary Fig. S6D). Similarly, pre-incubation of HT22^*cpe−/−*^ cells with CPE-WT, CPE-E342Q, or BDNF at 50 nM concentration alleviated glutamate-induced neurotoxicity (Supplementary Fig. S6E).

### CPE-E342Q mice lack CPE enzymatic activity and exhibit neuroendocrinological deficits

**Fig. 2:**
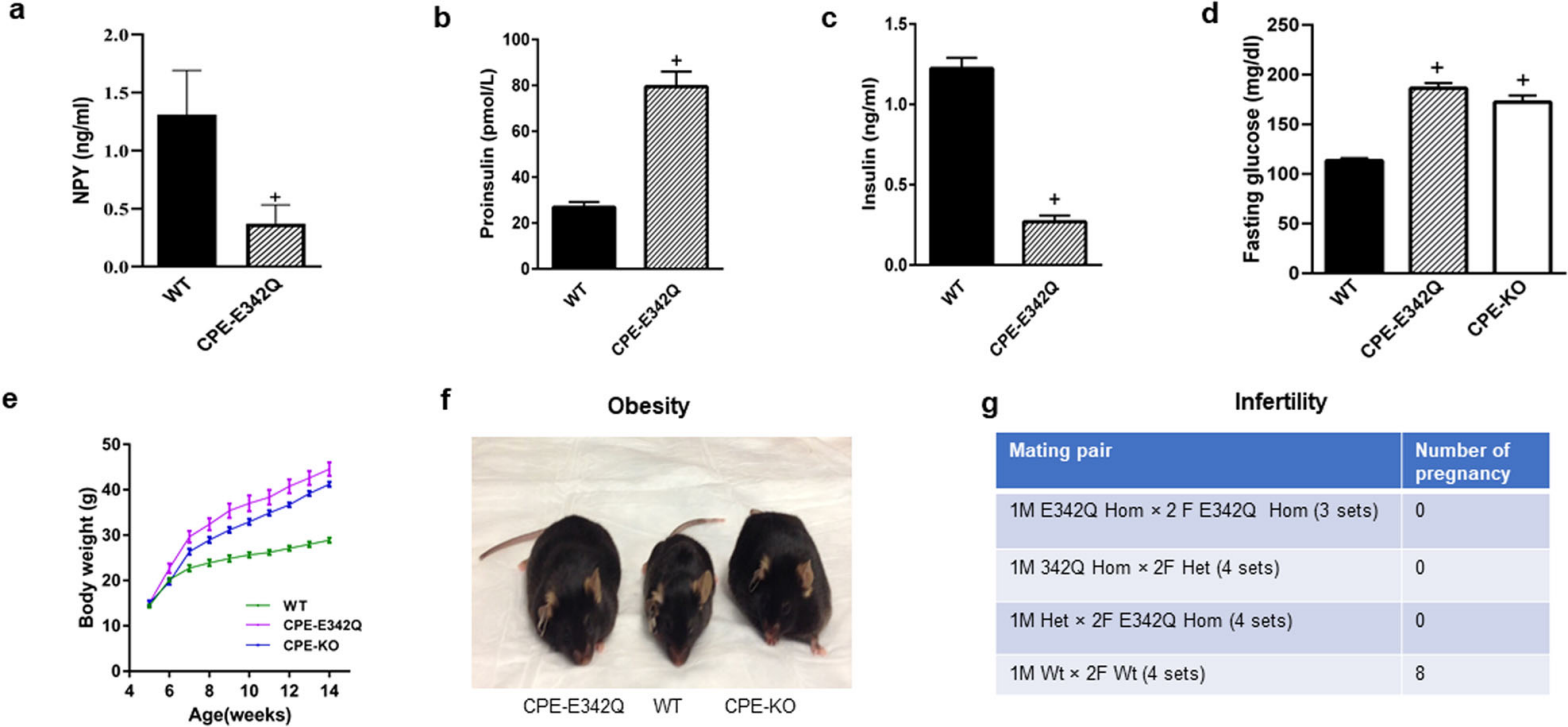
Neuroendocrinological deficits in CPE-E342Q mice due to lack of CPE enzymatic activity. **a** NPY analysis in CPE-E342Q mouse hypothalamus. Protein was extracted from WT and CPE-E342Q (*n* = 3 each) mouse hypothalamic extracts and NPY was measured using a mature NPY specific EIA kit. WT and CPE-E342Q (*n* = 3 each) hypothalamic tissue extracts were also separated on reverse phase HPLC column and fractions corresponding to NPY standard were analyzed by the NPY EIA kit. Bar graphs show collective result for both total and HPLC analyzed tissue samples (*n* = 6 mice). Note the 71.85% decrease in the level of mature NPY peptide in CPE-E342Q compared to WT mice, Mann Whitney’s non-parametric test, ^+^*p* = 0.0152, values are mean ± SEM. **b** Bar graphs show proinsulin in CPE-E342Q mice at 12 weeks of age was significantly increased in comparison with WT mice (WT = 26.9 ± 1.008 pmol/L, CPE-E342Q = 79.48 ± 2.94 pmol/L), Student’s *t* test, ^+^*p* < 0.0001 for CPE-E342Q mice compared with WT. The values are the mean ± SEM, *n* = 5 mice per group. **c** Bar graphs show insulin in CPE-E342Q mice at 12 weeks of age was significantly decreased in comparison with WT mice (WT = 1.223 ± 0.03 ng/ml, CPE-E342Q = 0.269 ± 0.017 ng/ml). Student’s *t* test, ^+^*p* < 0.0001 for CPE-E342Q mice compared with WT. The values are the mean ± SEM, *n* = 5 mice per group. **d** Bar graphs show fasting glucose levels were significantly higher in CPE-KO and CPE-E342Q mice in comparison with WT mice. One-way ANOVA analysis followed by Tukey’s post hoc multiple comparison test, [*F*_(2,45)_=92.62, *p* < 0.0001]. ^+^*p* < 0.0001 for both CPE-E342Q and CPE-KO mice compared with WT. The values are the mean ± SEM, *n* = 12 for CPE-KO, *n* = 12 for CPE-E342Q, *n* = 24 for WT. **e** Body weight of CPE-KO, CPE-E342Q, and WT mice. Weights of male and female WT, CPE-KO, CPE-E342Q mice were recorded from 5 to 14 week of age. The values are the mean ± SEM, *n* = 12 for CPE-KO, *n* = 12 for CPE-E342Q, *n* = 24 for WT. **f** Photograph showing the size of CPE-E342Q, WT and CPE-KO mice at 32 weeks of age. Note the obesity of CPE-E342Q and CPE-KO mice. **g** Fertility evaluation of CPE-E342Q mice compared with WT. Table shows CPE-E342Q homozygote mice are infertile. Hom = homozygote, Het = heterozygote, Wt = wild type. Mice were 6–9-week old.

Having demonstrated that CPE-E342Q lacks enzymatic activity, is properly trafficked and secreted from hippocampal neurons, and has neuroprotective activity, we generated a CPE-E342Q knock-in mouse (Supplementary Fig. S4) to determine if the neuroprotective activity of CPE can be replaced by an enzymatically inactive form, in vivo. To determine that the CPE-E342Q mice lack CPE enzymatic activity, we assayed pituitary extracts using dansyl-Phe-Ala-Arg as substrate. Supplementary Fig. S5B shows the virtual absence of CPE activity in the pituitary, similar to CPE-KO mice. To further confirm the lack of CPE activity in CPE-E342Q mice, levels of neuropeptide Y (NPY) and proinsulin/insulin in serum were analyzed. The level of NPY peptide in hypothalamus was 0.369 ng/ml in CPE-E342Q mice compared to 1.311 ng/ml in WT mice. (Fig. 2a). The diminished levels of NPY in the CPE-E342Q mice were very similar to CPE-KO mice^24^. CPE-E342Q mice showed increased serum proinsulin (Fig. 2b) and decreased insulin levels compared to WT mice (Fig. 2c). As a consequence of dysregulation of insulin, blood glucose level was increased in CPE-E342Q mice (Fig. 2d). Similar to CPE-KO mice, CPE-E342Q mice weighed significantly more than WT mice (Fig. 2e, f). Since CPE is involved in the processing of pro-GnRH, reproductive activity was evaluated. Breeding of heterozygote with homozygote, or homozygote with homozygote, males or females, failed to produce any pregnancies (Fig. 2g), indicating infertility, similar to CPE-KO mice^25^.

### CPE-E342Q mice show intact hippocampus, neurogenesis, and neurite integrity, after weaning stress, unlike CPE-KO mice

**Fig. 3:**
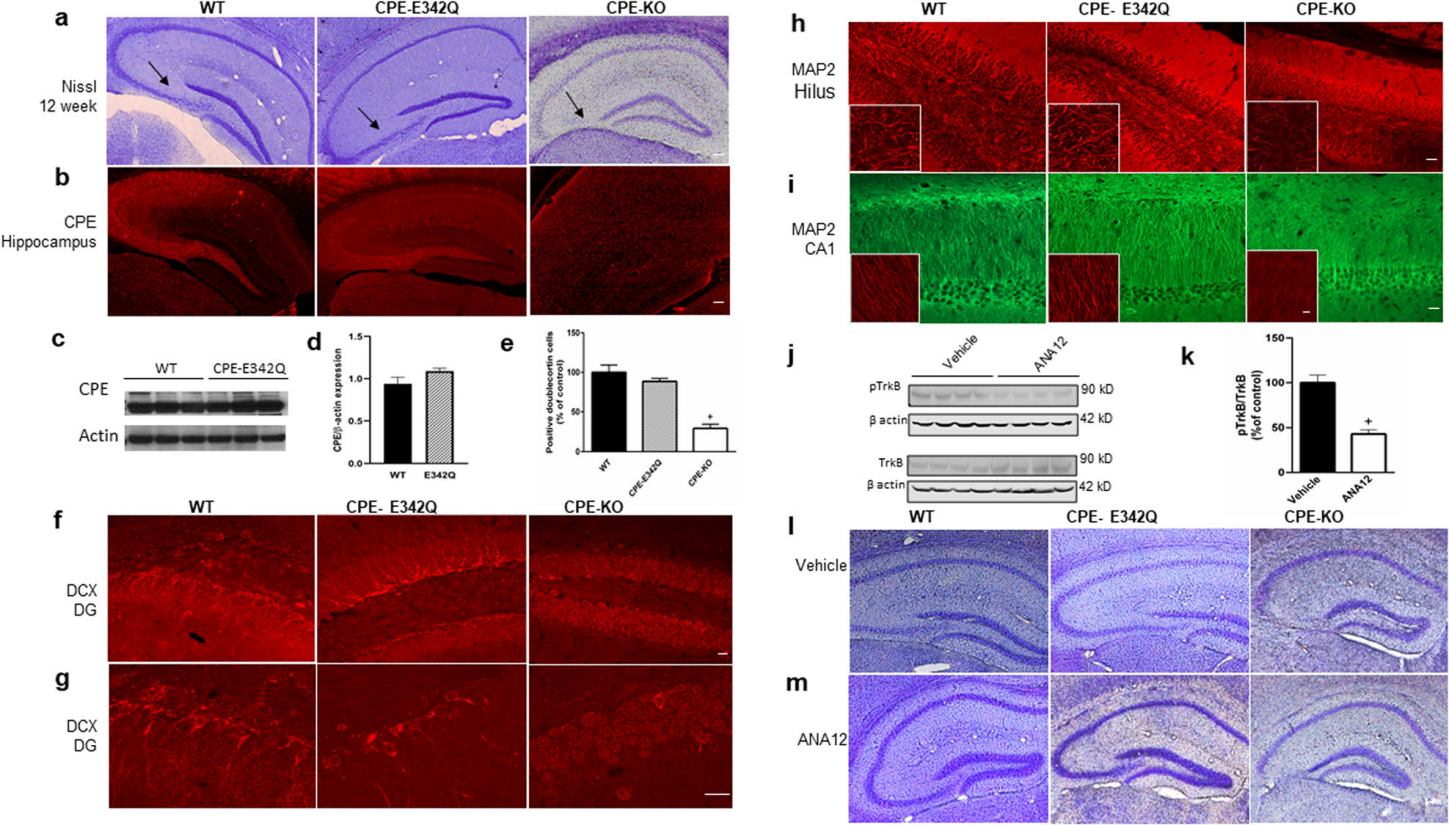
CPE-E342Q mice show no hippocampal neurodegeneration after weaning stress, unlike CPE-KO mice, and this effect was not blocked by TrkB inhibitor. **a** Representative sections of Nissl staining of coronal anterior hippocampus of 12-week-old WT, CPE-E342Q, and CPE-KO mice. CPE-E342Q mice showed an intact hippocampus similar to WT mice, however, CPE-KO mice exhibited complete degeneration of the CA3 region (see arrow). Scale bar=100 μm. *n* = 3 mice per genotype. **b** Immunohistochemistry of representative sections showing intense CPE staining in hippocampal CA1 and CA3 regions in WT and CPE-E342Q mice, but absence in CPE-KO mice. Sale bar=100 μm. *n* = 3 mice per genotype. **c**, **d** Western blot and quantification of expression of CPE in the hippocampus of WT and CPE-E342Q mice. Student’s *t* test, *p* = 0.149. *n* = 3 mice per genotype. Values are mean ± SEM. **e** Bar graphs showing % of DCX positive cells in the dentate gyrus of CPE-E342Q mice compared to WT and CPE-KO mice. Note the decreased number of DCX positive cells in CPE-KO but not CPE-342Q mice. One-way ANOVA analysis followed by Tukey’s post hoc multiple comparison test, [*F*_(2,6)_ = 43.32, *p* = 0.0003]. ^+^*p* = 0.0003 for CPE-KO mice compared with WT which was made =100%. *n* = 3 per each genotype. Values are mean ± SEM. **f**, **g** Representative confocal images captured at 20X and 60X, respectively, of doublecortin (DCX) immunofluorescence staining showing decreased hippocampal dentate gyrus (DG) neurogenesis in CPE-KO mice, but not in WT and CPE-E342Q mice. Scale bars in *F* = 20 μm and *G* = 20 μm. *n* = 3 mice per genotype. **h** MAP2 immunofluorescence staining images captured at 20X and 60X (left bottom insert) showing decreased MAP2 intensity in the hilus of CPE-KO mice, but not in WT and CPE-E342Q mice. *n* = 3 per genotype. Scale bar = 20 μm. **i** MAP2 immunofluorescence staining images captured at 20X and 60X (left bottom insert) showing decreased MAP2 intensity in the hippocampal CA1 region of CPE-KO mice, but not in WT and CPE-E342Q. *n* = 3 per genotype, Scale bar = 20 µm. **j**, **k** Western blot and bar graph of pTrkB and TrkB levels in the hippocampus of mice treated with ANA12 or vehicle. WT animal received ANA12 or vehicle treatment for 2 weeks and hippocampus was dissected to determine pTrkB/TrkB levels. Using this paradigm, ANA12 effectively decreased pTrkB/TrkB to 43.08% compared with vehicle-treated (control) mice. Student’s *t* test, ^+^*p* = 0.001 for ANA12 treatment compared with vehicle. Values are mean ± SEM, *n* = 4 mice/condition. **l**, **m** The effect of TrkB inhibitor, ANA12 on hippocampal CA3 region in CPE-WT, CPE-E342Q, and CPE-KO mice after weaning stress. Representative Nissl staining of sections of coronal anterior hippocampus of WT, E342Q, and CPE-KO mice at week 4 after ANA12 treatment. All mice were toe clipped within postnatal day 7 and received ANA12 i.p injection starting from postnatal day 14 for 14 days. Mice were weaned, tail clipped, and ear tagged at 3 weeks of age and sacrificed at week 4. *n* = 4 mice/condition, scale bar = 100 µm.

Nissl and immunohistochemistry analyses were carried out on 12-week-old CPE-E342Q, WT, and CPE-KO mice after the weaning stress paradigm at age 3 weeks. CPE immunohistochemistry (Fig. 3b) and Western blot (Fig. 3c, d) revealed that CPE and CPE-E342Q protein are highly expressed in the hippocampus, in the CA1 and CA3 regions of WT and CPE-E342Q mice, while completely absent in CPE-KO mice (Fig. 3a). Nissl staining of CPE-E342Q mouse brains revealed an intact hippocampus similar to WT mice (Fig. 3a); while CPE-KO mice showed complete degeneration of the hippocampal CA3 region (Fig. 3a). Similar results were obtained for 4–5-week-old mice (Supplementary Fig. S7A). Doublecortin (DCX) immunostaining revealed that neurogenesis in the hippocampal dentate gyrus of CPE-E342Q mice was similar to WT, but significantly decreased in CPE-KO mice (Fig. 3e–g). Immunostaining with anti-MAP2 to visualize neurite integrity showed MAP2 intensity in the hippocampal hilus and CA1 regions of CPE-E342Q mice was similar to WT mice (95.5% imaged at 20X and 100.98% imaged at 60x compared with WT in hilus; 99.55% imaged at 20X and 96.06% imaged at 60X compared with WT in CA1), but decreased in the CPE-KO mice (43.47% imaged at 20X and 42.22% imaged at 60X in hilus, and 41.16% imaged at 20X and 38.21% imaged at 60X in CA1 compared with WT, respectively) (Figs. 3h, i; S8A–D). However, these changes were not observed in the prefrontal cortex or hypothalamus of WT, CPE-KO or CPE-E342Q mice (Supplementary Figs. S7B, C; S8E, F).

### WT and CPE-E342Q mice show no CA3 degeneration with reduction of TrkB signaling after weaning stress

To determine whether TrkB signaling is required for the neuroprotection of CA3 neurons in vivo with the weaning stress paradigm, ANA12, a TrkB inhibitor, or vehicle, was administered i.p daily for 14 days beginning at week 2 to week 4 of age, to CPE-E342Q, WT and CPE-KO mice. Mice were subjected to the weaning paradigm at week 3 and sacrificed at week 4. ANA12 treatment inhibited phosphorylation of TrkB in the hippocampus by ~56.9% (Fig. 3j, k). However, these CPE-342Q mice showed no difference in hippocampal CA3 cytoarchitecture compared to WT mice after the weaning stress paradigm, suggesting that neuroprotection was not attenuated by reduction of BDNF-TrkB signaling (Fig. 3l, m).

### CPE-E342Q mice display normal learning and memory, but depressive-like behavior

**Fig. 4:**
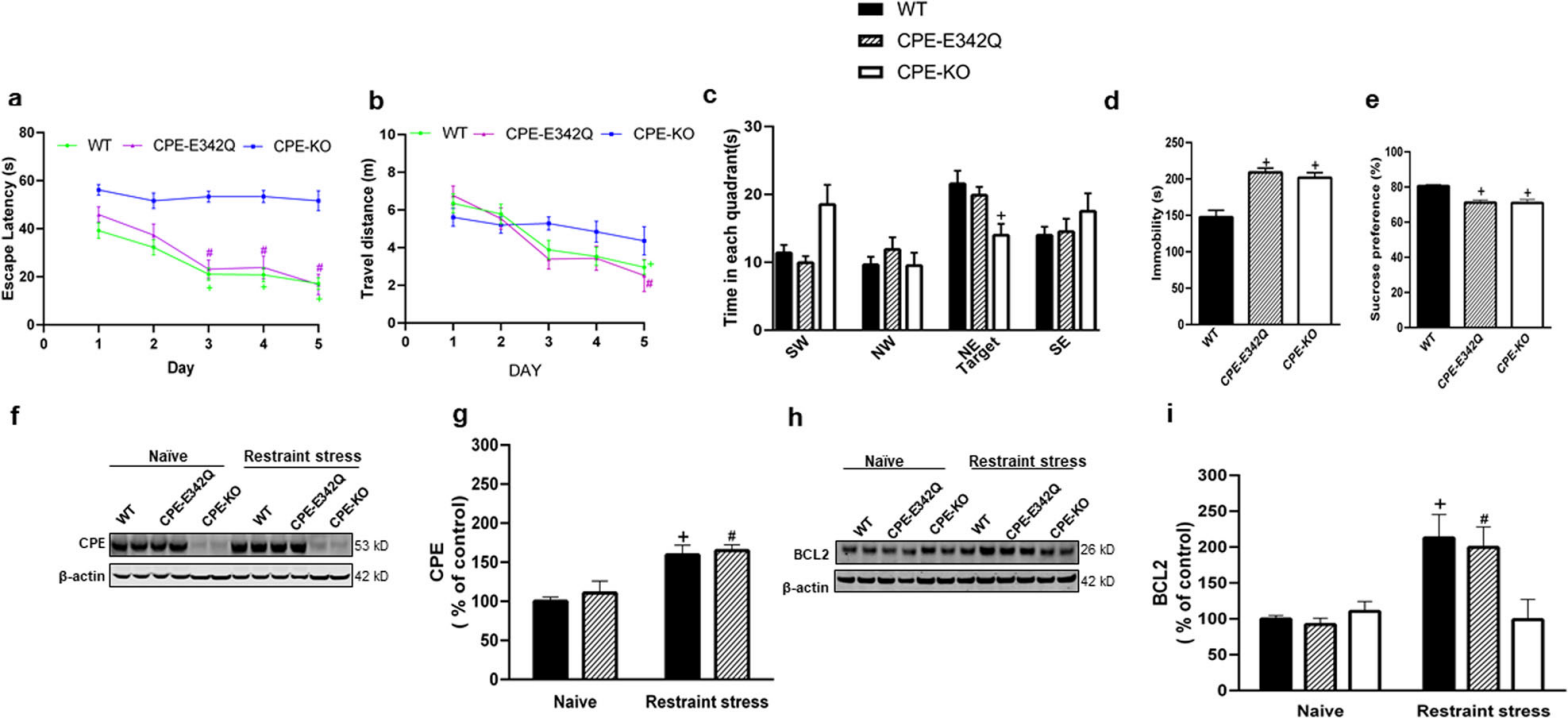
CPE-E342Q mice have normal cognitive function but display depressive-like behavior and demonstrate increased CPE and BCL2 after restraint stress. **a** Escape latency of WT, CPE-KO, and CPE-E342Q mice in Morris water maze test. WT and CPE-E342Q mice exhibited a normal learning acquisition curve during the 5-day test, and spatial memory; whereas CPE-KO mice demonstrated a deficit in spatial learning and memory. Two-way repeated ANOVA analysis followed by Tukey’s post hoc multiple comparison test. Day × Genotype: [*F*_(8,180)_ = 2.899, *p* = 0.0046]; Day: [*F*_(3.783,170.2)_ = 17.09, *p* < 0.0001]; Genotype: [*F*_(2,45)_ = 46.15, *p* < 0.0001]. Escape latency was decreased on day 3 (^+^*p* = 0.0043), day 4 (^+^*p* = 0.0031), and day 5 (^+^*p* < 0.0001) in WT and day 3 (^#^*p* = 0.0019), day 4 (^#^*p* = 0.0173), and day 5 (^#^*p* = 0.0036) in CPE-E342Q mice compared with day 1 respectively, but not in CPE-KO mice. *n* = 24 for WT, *n* = 12 for CPE-E342Q, *n* = 12 for CPE-KO. Values are mean ± SEM. **b** Travel distance during 5-day training of the water maze test. WT and CPE-E342Q showed no significant differences in travel distance during the 5-day training, although the CPE-KO mice tended to show a longer travel distance in days 3–5 of training compared to WT mice. Two-way repeated ANOVA analysis followed by Tukey’s post hoc multiple comparison test. Day × Genotype: [*F*_(8,180)_ = 1.702, *p* = 0.1007]; Day: [*F*_(3.611,162.5)_ = 15.40, *p* < 0.0001]; Genotype: [*F*_(2,45)_ = 1.148, *p* = 0.3265]. ^+^*p* = 0.0004 for WT day 5 compared with day 1; ^#^*p* = 0.014 for CPE-E342Q day 5 compared with day1. *n* = 24 for WT, *n* = 12 for CPE-E342Q, *n* = 12 for CPE-KO. Values are mean ± SEM. **c** Time spent in each quadrant of WT, CPE-KO, and CPE-E342Q mice in Morris water maze test. WT and CPE-E342Q mice spent more time in target quadrants than CPE-KO mice which spent about the same time in each quadrant. Two-way repeated ANOVA followed by Tukey’s post hoc multiple comparison test. Area × Genotype: [*F*_(6,135)_ = 2.986, *p* = 0.009]; Genotype: [*F*_(2,45)_ = 14.64, *p* < 0.0001]; Area: [*F*_(2.474,111.3)_ = 8.030, *p* = 0.0002]. ^+^*p* = 0.015 for CPE-KO compared with WT mice in NE target area. SW(Southwest), NW(Northwest), NE(Northeast), SE(Southeast). *n* = 24 for WT, *n* = 12 for CPE-E342Q, *n* = 12 for CPE-KO. Values are mean ± SEM. **d** Immobility time of WT, CPE-KO, and CPE-E342Q mice in forced-swim test. Both CPE-KO and CPE-E342Q mice displayed increased immobility in the forced-swim test, suggesting depressive-like behavior. One-way ANOVA followed by Tukey’s post hoc multiple comparison test. [*F*_(2,45)_ = 15.64, *p* < 0.0001]. ^+^*p* < 0.0001 for CPE-E342Q compared with WT; ^+^*p* = 0.0003 for CPE-KO compared with WT. *n* = 24 for WT, *n* = 12 for CPE-E342Q, *n* = 12 for CPE-KO. Values are mean ± SEM. **e** Sucrose preference of WT, CPE-KO, and CPE-E342Q mice. CPE-KO and CPE-E342Q mice both displayed decreased preference for sucrose, suggesting depressive-like behavior. One-way ANOVA analysis followed by Tukey’s post hoc multiple comparison test. [*F*_(2,45)_ = 18.77, *p* < 0.0001]. ^+^*p* < 0.0001 for both CPE-E342Q and CPE-KO compared with WT. *n* = 24 for WT, *n* = 12 for CPE-E342Q, *n* = 12 for CPE-KO. Values are mean ± SEM. **f** Representative Western blot of CPE in WT, CPE-E342Q, and CPE-KO (which showed no expression of CPE) and (**g**) quantification showing CPE protein level in the hippocampus of WT, and CPE-E342Q mice subjected to chronic restraint stress. Two-way ANOVA analysis followed by Tukey’s post hoc multiple comparison test. Genotype × Restraint stress: [*F*_(1,12)_ = 0.0566, *p* = 0.816]; Restraint stress: [*F*_(1,12)_ = 27.81, *p* = 0.0002]; Genotype: [*F*_(1,12)_ = 0.5668, *p* = 0.4660]. ^+^*p* = 0.01 for restraint stressed WT compared with naïve WT; ^#^*p* = 0.018 for restraint stressed CPE-E342Q, compared with naïve CPE-E342Q. *n* = 4 mice, values are mean ± SEM. **h** Representative western blot and (**i**) quantification showing BCL2 protein level in the hippocampus of WT, CPE-E342Q, and CPE-KO mice challenged to chronic restraint stress. Two-way ANOVA analysis followed by Tukey’s post hoc multiple comparison test. Genotype × Restraint stress: [*F*_(2,18)_ = 5.019, *p* = 0.0185]; Restraint stress: [*F*_(1,18)_ = 14.74, *p* = 0.0012]; Genotype: [*F*_(2,18)_ = 3.047, *p* = 0.0725]. ^+^*p* = 0.022 for restraint stressed WT compared with naïve WT; ^#^*p* = 0.031 for restraint stressed CPE-E342Q compared with naïve CPE-E342Q. *n* = 4 mice, values are mean ± SEM.

Cognition and depressive-like behavior were evaluated in 8–10-week-old CPE-E342Q, WT, and CPE-KO mice. Both CPE-KO and CPE-E342Q mice showed a significant decrease in travel distance (Supplementary Fig. S9A) and speed (Supplementary Fig. S9B) in the open field test compared to WT mice. In the Morris water maze test, the traveling distance (Fig. 4b) was similar for WT and CPE-E342Q mice, although CPE-KO tended to have a longer travel distance in days 3–5 of training than WT and CPE-E342Q mice (Fig. 4b). While CPE-KO mice displayed significant deficits in learning and memory in the Morris water maze test, CPE-E342Q mice exhibited normal memory and cognitive function (Fig. 4a, c). Elevated plus maze test did not reveal anxiety-like behavior in all these three genotypes (Supplementary Fig. S9C, D). However, CPE-E342Q mice showed depressive-like behavior indicated by the forced-swim test (Fig. 4d) and sucrose preference test (Fig. 4e) similar to CPE-KO mice, but different from WT mice.

### Restraint stress up-regulates CPE and BCL2 expression in the hippocampus of WT and CPE-E342Q, but not CPE-KO mice

To determine if CPE-E342Q mice respond in the same manner as WT mice to short term chronic stress, mice were restrained for 1 h per day for 7 days. Immediately after the 7th day of restraint stress, mice were sacrificed, and the hippocampi were analyzed. Figure 4f, g shows that the naïve CPE-E342Q mice express similar amounts of mutant CPE protein as CPE in WT mice. CPE and CPE-E342Q protein were significantly increased in the hippocampus after restraint stress (Fig. 4f, g). Furthermore, Fig. 4h, i shows that the pro-survival protein BCL2 was increased in CPE-E342Q hippocampus, similar to WT mice after restraint stress. CPE-KO mice, however, exhibited no increase in BCL2 expression in the hippocampus (Fig. 4h, i).

### CPE binds to HT22 cell surface membrane and activates ERK-BCL2 signaling independent of its enzyme activity

**Fig. 5:**
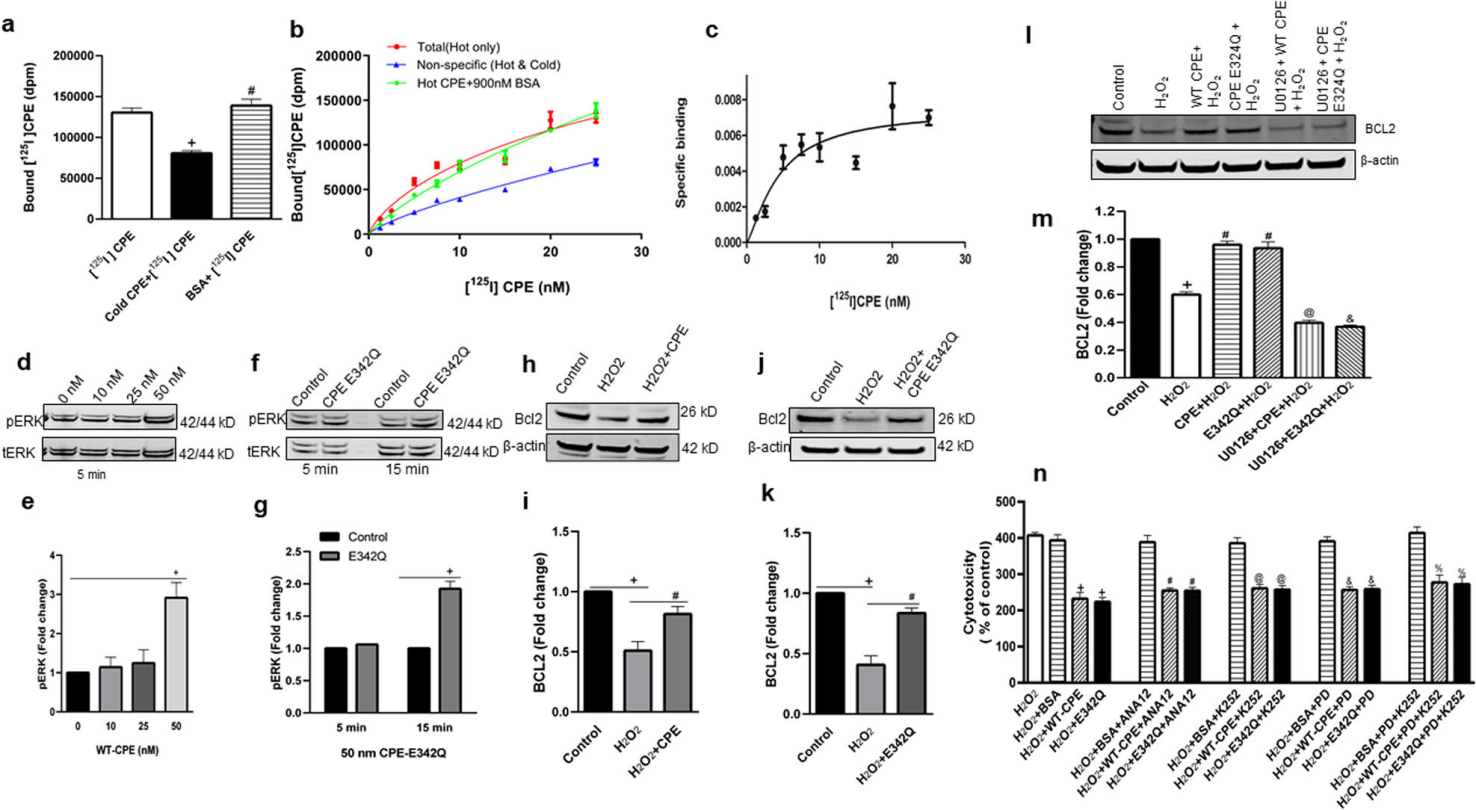
CPE binds to cell surface membrane and activates ERK-BCL2 signaling cascade, and its neuroprotective effect is tyrosine kinase independent. **a** [^125^I] CPE binds specifically to HT22 cell surface. HT22 cells were incubated with 25 nM [^125^I] radio-labeled CPE (hot) for 3 h on ice in serum-free binding medium. To demonstrate binding specificity, 900 nM non-radio-labeled CPE (cold) or bovine serum albumin (BSA) was co-incubated with hot CPE to compete and displace the hot CPE binding. The bar graphs show that the [^125^I] CPE binding to HT22 cell surface membrane was displaced by cold CPE but not by BSA. One-way ANOVA followed by Tukey’s post hoc multiple comparison test, [*F*_(2,6)_ = 29.11, *p* = 0.0008]. ^+^*p* = 0.002 for cold CPE + [^125^I] CPE compared with [^125^I] CPE (hot) treatment; ^#^*p* = 0.001 for BSA + [^125^I] CPE compared with CPE (cold)+ [^125^I] CPE treatment. The experiment was run in triplicates, values are mean ± SEM. **b** [^125^I] CPE binds HT22 cell surface in a saturated manner. HT22 cells were incubated with different concentrations (1.25–25 nM) of [^125^I] CPE (hot) with or without 900 nM cold CPE for 3 h on ice in serum-free binding medium. The non-specific binding was determined by measuring bound [^125^I] CPE in the presence of excess non-radio-labeled CPE (cold) with the [^125^I] CPE. Note that BSA did not displace total [^125^I] CPE binding. **c** Graph shows specific binding obtained by subtracting non-specific binding from total [^125^I] CPE binding. **d**–**g** CPE and CPE-E342Q activate ERK signaling in HT22^*cpe−/−*^ neuronal cells. **d** HT22 ^*cpe−/−*^ cells were treated with 0, 10, 25, and 50 nM WT-CPE for 5 min and pERK 1/2 were analyzed by western blotting. **e** Bar graphs showing the fold change in phosphorylation of ERK1/2 which was determined by normalization using tERK1/2 as an internal control. One-way ANOVA analysis followed by Tukey’s post hoc multiple comparison test, [*F*_(3, 8)_ = 9.830, *p* = 0.0046]. ^+^*p* = 0.0065 for 50 nM WT-CPE compared with control at 0 nM concentration. *N* = 3, values are mean ± SEM. **f** HT22 ^*cpe−/−*^ cells were treated with 50 nM CPE-E342Q for 5 min and 15 min and pERK 1/2 were analyzed by western blotting. **g** Bar graphs showing the fold change in phosphorylation of ERK1/2 which was determined by normalization using tERK1/2 as an internal control. One-way ANOVA analysis followed by Tukey’s post hoc multiple comparison test, [*F*_(3,8)_=61.33, *p* < 0.0001]. ^+^*p* < 0.0001 for CPE-E342Q treatment compared with control at 15 min. *N* = 3, values are mean ± SEM. **h**–**k** CPE and CPE-E342Q protect HT22^*cpe−/−*^ cells against oxidative stress by regulating BCL2 level. **h**, **j** Western blot analysis of BCL2 protein in HT22^*cpe−/−*^ cells which were treated with CPE-WT or CPE-E342Q respectively for 6 h followed by 100 μM H_2_O_2_ for 18–24 h. **i**, **k** Bar graphs showing quantification of BCL2 protein after normalization with β-actin which was used as a loading control. Results are expressed as fold change as compared to untreated controls. Similar to WT-CPE protein, CPE-E342Q was also able to prevent the H_2_O_2_ induced decrease in BCL2 level. For CPE-WT(**i**): one-way ANOVA analysis followed by Tukey’s post hoc multiple comparison test, [*F*_(2, 6)_ =19.37, *p* = 0.0024]. ^+^*p* = 0.002 for H_2_O_2_ compared to untreated control, ^#^*p* = 0.0201 for H_2_O_2_ + CPE compared to H_2_O_2_ treatment. *N* = 3, values are mean ± SEM. For CPE-E342Q (**k**): one-way ANOVA analysis followed by Tukey’s post hoc multiple comparison test, [*F*_(2, 6)_ = 38.69, *p* = 0.0004]. ^+^*p* = 0.0004 for H_2_O_2_ treatment compared to control, ^#^*p* = 0.002 for H_2_O_2_ + E342Q compared to H_2_O_2_ treatment. *N* = 3, values are mean ± SEM. **l, m** Effect of ERK inhibitor on CPE induced BCL2 expression. **l** Western blot analysis of BCL2 protein in HT22^*cpe−/−*^ cells which were treated with or without 5 μM MEK 1/2 inhibitor, U0126 for 30 min followed by treatment with 50 nM WT or E342Q recombinant CPE for 6 h. Cells were then treated with 100 μM H_2_O_2_ for the next 24 h. **m** Bar graphs showing quantification of BCL2 protein after normalization with β-actin which was used as a loading control. Results are expressed as fold decrease as compared to untreated controls. One-way ANOVA analysis followed by Tukey’s post hoc multiple comparison test, [*F*_(5,12)_ = 138.6, *p* < 0.0001]. ^+^*p* < 0.0001 for H_2_O_2_ compared to control, ^#^*p* < 0.0001 for both CPE + H_2_O_2_ and E342Q + H_2_O_2_ compared with H_2_O_2_ treatment, ^@^*p* < 0.0001 for U0126 + CPE + H_2_O_2_ compared with CPE + H_2_O_2_ treatment, ^&^*p* < 0.0001 for U0126 + E342Q + H_2_O_2_ compared with E342Q + H_2_O_2_ treatment_._ *N* = 3, values are mean ± SEM. **n** Neuroprotective effect of WT-CPE and CPE-E342Q against H_2_O_2_-induced cytotoxicity in mouse primary hippocampal neurons is independent of Trk receptor signaling. Bar graphs show that, 1 µM of Trk inhibitor, K-252a, or 100 µM of ANA12, a specific TrkB inhibitor, or 1 µM of FGFR1,2,3 inhibitor, PD166285 did not inhibit the neuroprotective effect of CPE and CPE-E342Q against H_2_O_2_-induced cytotoxicity assessed by LDH assay. One-way ANOVA analysis followed by Tukey’s post hoc multiple comparison test, [*F*_(15,32)_ = 26.93, *p* < 0.0001]. ^+^*p* < 0.0001 for H_2_O_2_ + WT-CPE and H_2_O_2_ + E342Q compared with H_2_O_2_ + BSA; ^#^*p* < 0.0001 for H_2_O_2_ + WT-CPE + ANA12 and H_2_O_2_ + E342Q + ANA12 compared with H_2_O_2_ + BSA + ANA12; ^@^*p* < 0.0001 for H_2_O_2_ + WT-CPE + K252 and H_2_O_2_ + E342Q + K252 compared with H_2_O_2_ + BSA + K252; ^&^*p* < 0.0001 for H_2_O_2_ + WT-CPE + PD and H_2_O_2_ + E342Q + PD compared with H_2_O_2_ + BSA + PD; ^%^*p* < 0.0001 for H_2_O_2_ + WT-CPE + PD + K252 and H_2_O_2_ + E342Q + PD + K252 compared with H_2_O_2_ + BSA + PD + K252 treatment. *N* = 3 independent experiments which were run in triplicate, values are mean ± SEM.

To determine the mechanism by which CPE mediates neuroprotection, we investigated CPE binding to HT22 cell surface. HT22 cells were incubated with [^125^I] CPE (hot) and with or without non-radio-labeled CPE (cold) to compete with the hot CPE binding. Figure 5a shows a decrease of total bound [^125^I] CPE in the presence of cold CPE indicating competition and specific binding of hot CPE. When BSA was added with [^125^I] CPE, there was no change in total [^125^I] CPE bound, verifying specificity of the binding. We then performed saturation-binding experiments using various concentrations of [^125^I] CPE (Fig. 5b) with and without cold CPE or BSA. BSA had no effect on total binding at various concentrations of [^125^I] CPE. Non-specific binding was established by the addition of excess cold CPE. Specific binding was obtained by subtracting non-specific binding from total binding (Fig. 5c). Saturation-binding data were analyzed by non-linear regression analysis using the PRISM program. The best-fit Kd value obtained was 4.37 nM for [^125^I] CPE. These results suggest that CPE binds specifically, and with high affinity, to cell surface receptors in hippocampal HT22 cell line. To gain some insight into whether the putative CPE-receptor could be a member of the tyrosine kinase receptor families, we tested the effect of several tyrosine kinase inhibitors on neuroprotection by CPE and CPE-E342Q against oxidative stress in mouse primary hippocampal neurons. These included ANA12 a specific inhibitor for TrkB,^26,27^; PD166285, a tyrosine kinase inhibitor for FGFR 1, 2, 3, and 4^28^ and K-252a, an inhibitor for Trk A, B, and C^29^. Figure 5n shows that none of these inhibitors blocked the neuroprotective effect of CPE or CPE-E342Q against H_2_O_2_-induced oxidative stress on these neurons, suggesting that the CPE-mediated neuroprotective effect is not dependent on receptor tyrosine kinase signaling.

Previously, CPE was shown to protect rat primary hippocampal neurons against H_2_O_2_-induced oxidative stress through activating the ERK-BCL2 signaling pathway^22^. To determine if CPE-E342Q activates the ERK pathway, we treated HT22 ^*cpe−/−*^ cells with CPE-E342Q and assayed for ERK phosphorylation. As a control, we treated HT22 ^*cpe−/−*^ cells with 0, 10, 25, and 50 nM CPE for 5 and 15 min. A significant increase (2.9-fold) in phosphorylation of ERK 1/2 was observed with 50 nM CPE after 5 min of treatment (Fig. 5d, e). Based on these results we treated HT22^*cpe−/−*^ cells with 50 nM CPE-E342Q recombinant protein for 5 and 15 min and found that CPE-E342Q increased ERK 1/2 phosphorylation between 5 and 15 min of treatment (Fig. 5f, g). We then determined if CPE-E342Q could prevent reduction in BCL2 level caused by H_2_O_2_. In HT22^*cpe−/−*^ cells, up to 50% reduction in BCL2 expression in H_2_O_2_ treated cells was observed compared to control. This reduction of BCL2 was prevented by both 50 nM CPE (Fig. 5h, i) and CPE-E342Q (Fig. 5j, k) added to media 6 h before H_2_O_2_ treatment. This effect of CPE and CPE-E342Q on BCL2 was inhibited by the ERK inhibitor, U0126 (Fig. 5l, m). Interestingly, the point mutation in CPE-E342Q rendered a small decrease in the rate of activation of ERK phosphorylation compared to CPE-WT. Nevertheless, the slightly slower activation of ERK did not affect the efficacy of the downstream neuroprotective activity of the molecule. These results indicate that CPE-E342Q, like CPE, protects hippocampal neurons from oxidative stress by activating ERK-BCL2 pathway.

## Discussion

It is well known that the human hippocampus is particularly sensitive to elevated levels of glucocorticoids associated with traumatic and psychosocial stress. Such stress if persistant can be associated with cognitive dysfunction and depression^30–32^. Studies in rodents have shown that various types of stress including restraint (immobilization), emotional and physical stress, as well as global ischemia can lead to dendritic atrophy of the CA3 neurons which is reversible, or to complete neuronal cell death depending on the severity and duration of the stress^1,6–8^. Although complicated, due to both excitatory and inhibitory inputs to the CA3 pyramidal neurons that can cause these changes, glutamate released from the mossy fibers that project to these neurons in response to glucocorticoid elevation during stress seems to be the major contributor to the neurotoxicity^1^. A key question is what neurotrophic factors might play a role in protecting these CA3 neurons from cell death during stress. BDNF released from the dentate gyrus granule cell mossy fibers on to the CA3 pyramidal neurons in response to glucocorticoid stimulation^1^ is thought to be a key player in protecting these neurons during stress. A small number of CA3 neurons do express BDNF^33^; however, the level of BDNF in CA3 neurons did not change with immobilization/restraint stress^15^. In contrast, after restraint stress in adult mice and global ischemia in rats, expression of NF-α1-CPE which has been shown to have neuroprotective activity in vitro^22^ was up-regulated in the CA3 neurons in mice and rats which did not show degeneration^8,34^. Additionally, CPE-KO mice after restraint stress showed an increase in expression of BAX, a pro-apoptotic protein in the hippocampus compared to WT mice^34^. Furthermore, the CA3 region was completely degenerated in NF-α1-CPE-KO mice after weaning combined with physical stress^11,13^, while emotional stress of weaning alone resulted in partial degeneration^13^. These studies indicate that NF-α1-CPE plays a very important role in protecting the CA3 neurons from death during stress. In contrast, conditional BDNF-KO mice did not show any anatomical differences in the CA3 region compared to WT mice, post weaning^35^. Several studies on various *bdnf*^*−/−*^ mouse models also showed that global BDNF deficiency in mice does not result in widespread neuronal cell death but is required for survival of only small neuronal populations such as subdivisions of the substantia nigra^36^ and noradrenergic neurons in pontine nucleus^37^, but not in the hippocampus. An extensive study of a BDNF deficient model which can survive for 8 months, revealed that BDNF is not a major survival factor for most neurons in the brain, except a specific area, the striatum. At 8 weeks of age, post-weaning stress, these BDNF deficient mice exhibited no change in the growth of the hippocampus and only minimal changes in hippocampal pyramidal CA1 neuronal dendrites were observed^38^. Overall, studies on *bdnf*^*−/−*^ mice reported in the literature suggest that BDNF does not play a major role in the survival of hippocampal neurons.

Here we have investigated the relative importance of NF-α1-CPE versus BDNF in protecting the CA3 neurons from dying during severe stress in a paradigm which included a combination of emotional (maternal separation) and physical stress (ear tagging and tail clipping for genotyping) occurring at weaning in mice. We demonstrated that WT and NF-α1-CPE-KO mice at 3 weeks of age showed a hippocampus with normal cytoarchitecture. However, following the weaning stress paradigm at 3 weeks of age, the NF-α1-CPE-KO mice, but not the WT mice showed complete degeneration of the hippocampal CA3 region by 4 weeks of age. Analysis of BDNF and pTrkB in the hippocampus of WT and KO mice at 3 weeks of age revealed higher levels, and at 4 weeks, similar levels of both these proteins in the NF-α1-CPE-KO mice compared to WT controls. This result indicated that despite the expression of elevated or similar levels of BDNF-pTrkB, and having other growth factors (NGF, NT3, GDNF) in the hippocampus at levels similar to WT mice, NF-α1-CPE-KO mice, exhibited complete CA3 neurodegeneration with severe stress. Furthermore, WT mice treated with ANA12, a specific TrkB inhibitor which inhibited TrkB phosphorylation by ~57% did not result in any observable degeneration of the CA3 region after the weaning stress paradigm. Studies have shown that even a lesser inhibition of TrkB phosphorylation had significant effects on anxiety- and depression-like behaviors^26^. All these data taken together indicate that the BDNF-pTrkB signaling system does not play as critical a role as NF-α1-CPE, in preventing the death of CA3 neurons after severe stress, but is more important in milder stress-induced neuronal plasticity^15^.

We then determined if stress-induced CA3 degeneration can be prevented in knock-in mice expressing an enzymatically inactive form of CPE, CPE-E342Q, in the absence of NF-α1-CPE, which would otherwise be vulnerable to degeneration as in the CPE-KO mice. The CPE-E342Q mice exhibited poor prohormone processing as exemplified by decreased serum levels of insulin and elevated levels of proinsulin, leading to high circulating glucose and diabetes. Similarly, these mice also had other endocrinological deficits such as obesity and infertility as observed in CPE-KO mice^39^. As expected, these mice had diminished levels of Neuropeptide Y in the hypothalamus similar to NF-α1-CPE-KO mice^24^. However, these CPE-E342Q mice showed no evidence of degeneration of the hippocampal CA3 region after the weaning stress paradigm compared to NF-α1-CPE-KO mice which exhibited complete degeneration. MAP2 immunostaining for neurites indicated no difference in neurite integrity in the hippocampal CA1 region or hilus of the dentate gyrus similar to WT mice after weaning stress, but NF-α1-CPE-KO mice showed decreased neurite integrity probably due to atrophy of neurites in those regions. The neuroprotective effects of NF-α1-CPE seem to be most evident in the hippocampal CA3 region since the prefrontal cortex and hypothalamus showed no difference in neurite integrity between WT, CPE-E342Q, and NF-α1-CPE-KO mice. Moreover, there was normal neurogenesis in the dentate gyrus of CPE-E342Q mice similar to WT mice, whereas NF-α1-CPE-KO mice showed significantly decreased neurogenesis. As well, the CPE-E342Q protein and the mitochondrial pro-survival protein, BCL2, were also increased in the hippocampus after restraint stress of these mice, similar to WT mice. These data show that CPE-E342Q was able to replace NF-α1-CPE in mice to prevent cell death of the CA3 neurons and degeneration of neurites of the CA1 and hilus neurons, after the weaning stress paradigm. Additionally, CPE-E342Q mice were able to maintain neurogenesis in the subgranular zone of the dentate gyrus. Moreover, the CPE-E342Q mice, treated with ANA12, the TrkB inhibitor showed no CA3 neurodegeneration after the weaning stress paradigm. Given that these mice show no morphological changes in the hippocampal CA3 neurons that drive cognitive function^40^, it was not surprising that they showed normal spatial learning and memory in the water maze test. These results indicate that prevention of neurodegeneration and cognitive decline from severe stress in mice can be mediated by CPE-E342Q, and therefore NF-α1-CPE’s neuroprotective action is trophic and independent of its enzymatic activity.

Interestingly, these mice showed depressive-like behavior despite having normal neurogenesis. This model thus illustrates that neurogenesis is necessary, but alone may not be sufficient to prevent depressive-like behavior, as suggested by others^41^, and that other players are involved. Perhaps the neuropeptides that are lacking in these animals, unlike the WT mice, may account for the depressive-like behavior.

Cell biological studies showed that recombinant CPE-E342Q at 25 or 50 nM protected HT22^*cpe−/−*^ cells against H_2_O_2_-induced oxidative stress and glutamate-induced neurotoxicity, similar to WT-CPE. In organotypic hippocampal slice cultures of NF-α1-CPE-KO mice, the kainic acid treatment caused severe cell death of the CA3 neurons, whereas WT mice showed minimal degeneration. These findings support a strong neuroprotective role of NF-α1-CPE against glutamate-induced cell death of the CA3 neurons.

Activation of the signal transduction pathway by NF-α1-CPE would require its binding to a receptor. Studies using [^125^I] CPE showed binding to a putative receptor on the surface of HT22 cells in a specific and saturable manner with a Kd = 4.37 nM. None of the tyrosine kinase receptor inhibitors, ANA12, K-252a, or PD166285 abolished the neuroprotective effect in hippocampal neurons in culture when challenged with H_2_O_2._ Both NF-α1-CPE and CPE-E342Q binding to HT22^*cpe*−/−^ cells activated ERK phosphorylation and mitochondrial pro-survival protein, BCL2 expression to mediate neuroprotection, indicating the non-enzymatic role of NF-α1-CPE in signal transduction. Interestingly, applied recombinant NF-α1-CPE and CPE-E342Q also have an effect on enhancing mitochondrial ATP production observed in a preosteoblastic U33 cell line, and this potentially may be yet another mechanism that NF-α1-CPE could contribute to promoting cell survival^42^.

In summary, our findings indicate that NF-α1-CPE is a more critical trophic factor than BDNF for the prevention of CA3 neuronal cell death and cognitive decline with severe stress in mice. In vivo, when CA3 neurons are excited by glucocorticoid-induced glutamate release during stress, they secrete NF-α1-CPE which then acts in an autocrine-paracrine manner to mediate neuroprotection through binding to a putative receptor to induce the ERK-BCL2 signaling cascade. Additionally, NF-α1-CPE expression is up-regulated by glucocorticoid through GREs in its promoter^34^. Protecting these hippocampal CA3 neurons which drive learning and memory functions^40,43^ by NF-α1-CPE is key to preventing the cognitive dysfunction associated with severe stress. The potent neuroprotective activity of NF-α1-CPE, or the PPARγ agonist, rosiglitazone which up-regulates NF-α1-CPE expression in mouse hippocampus^12,44^, renders them as potentially useful therapeutic agents for treating neurodegenerative diseases as suggested by a pilot clinical study^45^.

## Supplementary information

Supplemental information

Supplemental information
