# Supplementary material for: Neurotrophic factor-α1, a novel tropin is critical for the prevention of stress-induced hippocampal CA3 cell death and cognitive dysfunction in mice: comparison to BDNF
Source: Transl Psychiatry. 2021 Jan 7;11:24. doi: 10.1038/s41398-020-01112-w (PMC7791060; doi:10.1038/s41398-020-01112-w)
Supplement: Supplementary file 1 — Supplemental information [file 41398_2020_1112_MOESM1_ESM.docx]

**Supplementary information for**

**Neurotrophic factor-α1, a novel tropin is critical for the prevention of stress-induced hippocampal CA3 cell death and cognitive dysfunction in mice: comparison to BDNF**

Lan Xiao, Vinay Kumar Sharma, Leila Toulabi, Xuyu Yang, Cheol Lee, Daniel Abebe, Areg Peltekian, Irina Arnaoutova, Hong Lou and Y. Peng Loh*

Section on Cellular Neurobiology, *Eunice Kennedy Shriver* National Institute of Child Health and Human Development, National Institutes of Health, Bethesda, MD 20892, USA

*Corresponding author:

Dr. Y. Peng Loh,

Section on Cellular Neurobiology,

49, Convent Drive, Bldg 49, Rm 6A-10,

NICHD, NIH, Bethesda, Md. 20892, USA.

Email: [lohp@mail.nih.gov](mailto:lohp@mail.nih.gov)

Tel: (301) 496-3239 Fax: (301) 496-9938

**This file includes:**

Supplementary Methods

Supplementary Figures S1 to S9

Supplementary References

**SUPPLEMENTARY METHODS**

**Generation of the HT22*^cpe-/-^*  cells**

HT22 cells is a mouse immortalized hippocampal cell line, subcloned from the HT-4 cell

line ^1^ and is a model neuronal cell system to study glutamate-induced toxicity. Endogenous CPE expression was knocked out in HT22 cells by means of non-homologous end-joining that occurs after introduction of gRNA/SpCas9-mediated double-strand DNA breaks. To achieve this, two gRNAs that correspond to exon1 of *mCPE* gene were co-transfected with a plasmid that bears resistance to neomycin into the HT22 cell line, using ViaFect reagent (Promega, catalogue #E4981) and G418-resistant clones were isolated. Genomic DNA from these clones were purified and analyzed by PCR using primers that anneal ~ 800 bp outside of the putative gRNA-cleavage points. PCR fragments were cloned into pJet vector using CloneJET PCR Cloning Kit (ThermoFisher Scientific, catalog# K1231) and 8-10 clones of each transformant were sequenced to verify breaks in all CPE alleles. Western blots were then carried out to screen for clones that did not express CPE. One clone was selected and named HT22*^cpe-/-^*. The gRNAs were cloned into px330 plasmid (Addgene, <https://www.addgene.org/42230/>) that also expresses codon-optimized SpCas9.

Guide RNA 1^st^:

caccgCCGTCAGCAGCCACCCGC

aaacGCGGGTGGCTGCTGACGGc

Guide RNA 2^nd^:

caccGTGTGCCGCGCTGGTGGC

aaacGCCACCAGCGCGGCACAC

**Stimulated secretion experiments**

Stimulated secretion studies were performed as previously described ^2^. HT22*^cpe-/-^* cells were transfected with empty vector or vector carrying CPE-WT or CPE-E342Q for 48 h and then incubated for 30-min with basal medium DMEM (Gibco, Waltham, MA), followed by 15-min incubation with stimulation medium supplemented with 50 mM KCI (Gibco, Waltham, MA). The cells and media from incubations were collected for Western blot.

**Enzymatic activity assay for WT-CPE and CPE-E342Q**

For *in vivo* experiments, total proteins from mouse hypothalamus and pituitary gland (WT, CPE-E342Q and CPE-KO, n=3) were extracted in T-PER protein extraction reagent (Thermo scientific Waltham, MA) containing protease inhibitor cocktail, PMSF and 0.1% Triton X-100, using silica beads. Tissue extracts were centrifuged at 17000xg for 30 min. and supernatants were transferred to a new tube for enzyme assay. Supernatant containing 500 ng proteins was incubated with 100 μl of dansyl-Phe-Ala-Arg substrate (0.5 mM) in 100 mM sodium acetate buffer (pH 5.5) for 16 h at 37°C. Similar experiments in the presence of 1 mM CoCl_2_ or 1 mM GEMSA (CPE inhibitor, Abcam USA) were also performed, and fluorescence was recorded at excitation (360/40 nm filter) and emission (528/20nm filter). Enzymatic activity of recombinant CPE-E342Q (*in vitro*) was also tested where 100 ng purified proteins (CPE-WT or CPE-E342Q) were incubated with dansyl substrate in the presence of CoCl_2_ and GEMSA. These experiments were performed in triplicates.

**Production of recombinant mouse CPE-WT and CPE-E342Q**

mWT-CPE and mCPE-E342Q were custom synthesized (GenScript, Piscataway,NJ) and are his-tagged proteins without the propeptide. They both were produced in human HEK293 cells and expressed with Vector pTT5 carrying the mWT CPE or mCPE-E342Q construct. Protein was obtained from supernatant of cell culture medium and purified by His Trapp^TM^FF Crude and HiLoad16/600 superdex x 200pg procedure. The purity of each of the proteins was evaluated by SDS PAGE/Coomassie blue stain and Western blot. Coomassie blue stain and Western blot revealed one band only of ~53kD under reducing conditions for CPE-E342Q and no other bands, and for CPE-WT, besides the major 53kD band, there were 2 additional very minor bands with Coomassie blue stain and one very minor band in the Western blot which we identified as a CPE breakdown product. Genscript described the preparation as >95% pure.

**Transfection or treatment of HT22*^cpe-/-^* cells with CPE-WT or CPE-E342Q followed by treatment with and without H_2_O_2_ or glutamate and LDH assay.**

For studies of the neuroprotective effect of expressed CPE in HT22*^cpe-/-^*  cells, the cells were transfected with plasmids that express CPE-WT or CPE-E342Q for 48h, and then challenged with 100 µM H_2_O_2_ for 6h. Cell cytotoxicity was evaluated by LDH assay and the cell lysates were analyzed by Western blot. For studies of the neuroprotective effect of recombinant CPE against H_2_O_2_ cytotoxicity, HT22*^cpe-/-^* cells were treated with 10, 25, 50 nM BSA, CPE-WT or CPE-E342Q for 24h and then challenged with 100 µM H_2_O_2_ for 6h. Cell cytotoxicity was evaluated by LDH release assay. For neuroprotective effect of recombinant CPE and BDNF against glutamate-induced neurotoxicity, HT22*^cpe-/-^* cells were treated with 50nM recombinant CPE-WT, CPE-E342Q or BDNF (PeproTech, Rocky Hill, NJ) 24h and then challenged with 40 µM glutamate (Sigma-Aldrich, St. Louis, MO) for 24h. Cell toxicity was evaluated with LDH assay.

**Generation of the CPE-E342Q knock in mouse**

To generate a knock-in (KI) mouse model carrying E342Q point mutation that results in the change from Glutamic acid (E) to Glutamine (Q) at the 342th amino acid in exon 6 in mouse *mCpe* gene, a strategy combining CRISPR/Cas9 and microinjection to the embryo was used. A guide RNA mMCpe.g5 targeting nucleotide sequence (5’- TAGCTGTGAGAAGTTCCCACCGG -3’) encoding E342 of mouse mCpe gene and a single stranded oligo deoxylnucleotide (ssODN) serving as a donor during the Homology-Directed-Repair (HDR) process were designed. The mixture containing mMCpe.g5 (gRNA), ssODN donor and Cas-9 mRNA was injected into the cytoplasm of C57BL/6 (B6) embryos. New mice born from the microinjection were screened by PCR and sequencing. Four heterozygous founders carrying the E342Q mutation were confirmed by genotyping. A three-generation breeding scheme were performed to generate F2 homozygous mice carrying E342Q mutation. The gene editing strategy is illustrated in Supplementary Fig. S3. These mice were generated under a contract with Applied Stem Cell (Milpitas, CA).

**Antibodies for Western blot**

Monoclonal mouse anti-CPE antibody (BD Biosciences, Franklin Lakes, NJ,Cat.#

610758), monoclonal mouse BCL2 antibody (1:3000,Cell signaling, Danvers, MA, Cat. #15071), Rabbit NGF antibody (1:3000, Abcam, Cambridge, MA, Cat.#18956), BDNF antibody (1:500, Thermo Scientific, Waltham, MA, Cat.# PA1-18357 ; 1:1000, Abcam, Cambridge, MA Cat.#108319 ) , rabbit GDNF antibody (1:3000, Abcam, Cambridge, MA, Cat.#18956), NT-3 antibody (1:3000, Abcam, Cambridge, MA, Cat.#53685), rabbit TrkB antibody (1:3000, Abcam, Cat.#18987 or 1:1000, Cell signaling,Cat. #4603 ), rabbit phospho-TrkB antibody (Tyr816) in Fig.3J,K (1:2000, Sigma-Aldrich, St. Louis, MO, Cat.#1381) or (Tyr706/707) (1:1000, Cell signaling, Cat.#4621) in Fig.1 J,K , monoclonal mouse or rabbit β-actin antibody (1:5000, Cell signaling,Cat.#3700; #4970) and GAPDH antibody (1:2000, Santa Cruz, Cat.# sc-32233) were used. The protein expression level for each sample was normalized to *β*-actin or GAPDH.

**Behavioral Studies.**

For behavioral tests, M=16,F=8 for WT;M=8,F=4 for CPE-KO; M=7,F=5 for CPE-E342Q. M=male; F=female. The illumination at the floor of the arena or maze was about 30-35 lux. Any mice with infections, significantly decreased weight or any obvious conditions were excluded from experiments.

*Open field*

To evaluate the locomotor activity, WT(n=24), CPE-E342Q (n=12) and CPE-KO mice (n=12) were placed into the open field for 1 h. Moving distance and speed were monitored and analyzed by ANY-maze software (ANY-maze, Wood Dale, IL).

*Elevated plus maze*

The elevated plus maze was conducted as previously described ^3^. The apparatus is made of polypropylene and comprises two open and two close arms that extended from a central platform. Mice were individually placed in the central area facing open arm and allowed to freely explore the apparatus for 5 min. The time and entries into arms were video recorded and analyzed with ANY-maze software.

*Morris Water Maze*

Morris water maze was used to study spatial learning and memory as previously described ^4^. WT (n=24), CPE-KO (n=12) and CPE-E342Q (n=12) mice at 8-10 weeks of age were used. The test consists of a 5-day hidden platform training and 1-day probe test. Test was performed in a circular pool (diameter of 1m) filled with water and nontoxic white paint. Video tracking and navigational parameters were analyzed with Any Maze software (ANY-maze, Wood Dale, IL). On day 1, the mouse was placed in the pool facing towards the wall. There are four trials each day and mice were placed in a new quadrant on each trial. The hidden platform was put in the same position for all four trials. Mice would search for the platform for 1 min and were then placed on the platform for 30 seconds before being removed. If mice did not find the hidden platform, they were guided to the platform and allowed to sit on it for 30 seconds. Escape latency, the time for mice to find the hidden platform was recorded for five days. Twenty four hours after the last training session, the hidden platform was removed, and the mice were allowed to explore the pool for 1 min. The time mice spent in each quadrant was recorded and analyzed by ANY-maze software (ANY-maze, Wood Dale, IL).

*Forced-swim test*

The forced-swim test was used to evaluate depressive-like behavior and was conducted as previously described ^5^. The apparatus was a transparent cylinder container filled with water at 23 °C. Swimming behavior was recorded for 6 min and immobility in the last 4 min was reanalyzed by ANY-maze software. Immobility time is defined as the time that the animal spent floating or engaged in minimal activity to keep their heads above the water.

*Sucrose preference test*

Sucrose preference test was used to evaluate depressive-like behaviors and was performed as previously described ^5^. Mice were first habituated to the presence of two bottles that contain sterile water for 5 days and then allowed to choose to drink either 1% sucrose solution or sterile water for 4 days. New bottles of fresh sucrose and water were used every day to avoid microbiological contamination. To reduce the confounding concerns associated with side bias, the positions of the two bottles were switched daily. Sucrose preference was calculated as a percentage of (the volume of sucrose intake)/ (volume of sterile water+ sucrose).

*Restraint stress paradigm for mice*

This procedure is a frequently used stressor that stimulates the hypothalamus-pituitary-adrenal axis and increases plasma corticosterone levels. The restraint stress was conducted by confining a mouse in a plastic tube 1 hour per day for 7 days as described previously ^6^. On day 7 after the last restraint stress, mice were sacrificed by cervical dislocation for biochemical studies.

**Quantitative Real-time PCR**

Total RNA was isolated from freshly dissected hippocampi using Trizol reagent (Invitrogen, Carlsbad, CA,). Equal quantities (100 ng) of total RNA were then reverse transcribed to obtain cDNA by using iScript kit (BioRad, Hercules, CA). qRT-PCR analysis measuring SYBR Green incorporation (Roche, Basel, Switzerland) in a Roche Light Cycler 480 PCR machine. Gapdh was used as a reference gene. Samples were duplicated for each run and PCR were repeated twice. Primers used are Bdnf-F: 5’-AGCTGAGCGTGTGTGACAGT-3’, Bdnf-R: 5’-ACCCATGGGATTACACTTGG-3’, TrkB-F: 5'-AAGGACTTTCATCGGGAAGCTG-3’, TrkB-R: 5'-TCGCCCTCCACACAGACAC-3’.

**Neuropeptide analysis**

To assay NPY, mouse hypothalami from WT, CPE-E342Q and CPE-KO animals were homogenized in 100 μl of 0.25% glacial acetic acid in the presence of protease inhibitor cocktail. These homogenates were heated at 95°C for 10 min followed by centrifugation at 17,000 rpm for 30 min. The supernatants were saved, and pellets were re-extracted with 100 μl of 0.25% acetic acid and combined with the first supernatant and lyophilized. The samples from WT and CPE-E342Q hypothalamic tissues were assayed for NPY using an EIA kit (Phoenix Pharmaceuticals, Burlingame, CA) for the mature peptide. NPY-IR WT and CPE-E342Q tissue extract were also analyzed by HPLC followed by EIA. The samples were separated by HPLC on a Jupiter C18 column (Phenomenex, Torrance, CA). The column was equilibrated in 20% buffer B (80% acetonitrile in 0.1% TFA) and 80% buffer A (0.1% TFA) ^7^. The gradient went from 20% buffer B to 80% buffer B in 35 min. Eluted fractions (no. 26–30 corresponding to elution of NPY standard) were lyophilized and reconstituted in EIA buffer and assayed for mature NPY. Five μg NPY-amide standard peptide (Phoenix Pharmaceuticals, Burlingame, CA) was applied to the column and eluted in Fraction 28 as observed by OD at 214 nm. NPY values assayed from both total tissue extract or after HPLC were in a similar range and therefore the data were combined.

**Serum insulin, proinsulin and blood glucose measurements**

Serum insulin and proinsulin were measured with Ultra Sensitive Mouse Insulin ELISA kit (Crystal Chem, Elk Grove Village, IL) and rat/mouse proinsulin Elisa kit (Creative diagnostic, Shirley, NY) according to manufacturer’s instructions, respectively. Blood glucose levels were measured with a glucometer.

**Evaluation of fertility**

Fertility was evaluated by pairing E342Q homozygote male and females (3 sets), E342Q homozygote male or female with E342Q heterozygote female or male (4 sets respectively), compared with WT male and female (4 sets) for 30 days, or until the female demonstrated detectable signs of pregnancy, which ever happened first.

**Transfection or treatment of HT22*^cpe-/-^* cells with CPE-WT or CPE-E342Q followed by treatment with and without H_2_O_2_ or glutamate and LDH assay.**

To study the neuroprotective effect of expressed CPE in HT22*^cpe-/-^*  cells or recombinant CPE, cells were transfected or treated with recombinant CPE, challenged with 100 µM H_2_O_2_ or 40 µM glutamate and LDH assay performed as described in Supplementary Methods.

**LDH release assay for cell toxicity**

Cell toxicity was assessed by the amount of LDH released using a CytoTox 96 Non-radioactive cytotoxicity assay kit according to manufacturer’s instruction (Promega, Madison, WI).

**^125^I CPE radio-ligand binding to HT22 cells**

Competitive and specific binding studies were performed using ^125^I CPE and HT22 cells.

Twenty-four hours prior to the binding assay, 5x10^5^ cells/well HT22 cells were seeded in a 12-well plate in triplicates for each data point. Cells were at confluency (1 x10^6^ per well) at the time of binding assay. CPE was labelled with ^125^I according to Pierce Iodination Protocol at Imaging Probe Development Center (IPDC, NIH). The probe was purified by a mini PD10 column and the specific activity of ^125^I CPE was 3218Ci/mmol. For the titration experiments, the following concentrations ^125^I CPE: 25, 20,15, 10, 7.5, 5, 2.5, 1. 25 (nM) were used. Cells were washed three times with serum-free binding medium (serum-free DMEM) and then incubated with radio-labeled CPE (“hot”) for 3h. on ice in serum-free binding medium. For competitive binding experiments 900 nM of unlabeled CPE (“cold”) was also included in each experiment. After incubation, cells were washed three times with 1ml of serum-free DMEM and then lysed in 1 ml of 0.5 M NaOH and counted in a gamma-counter (Packard Cobra II, GMI, Ramsey, MN). To verify specificity, binding was carried out with equivalent molar amounts of unlabeled pure bovine serum albumin, a protein close in molecular mass to that of CPE.

**Treatment of primary mouse hippocampal neurons with CPE-WT or CPE-E342Q with and without H_2_O_2_ and other inhibitors.**

Primary hippocampal neurons were grown in DMEM (Gibco, Waltham, MA) supplemented with 10% FBS (Atlanta Biologicals, Flowery Branch, GA) and replaced with Neurobasal medium (Gibco, Waltham, MA) with 2% B27 (Invitrogen, Carlsbad, CA). Medium was changed twice a week and used in experiments after 5-7 days DIV. Primary cultured hippocampal neurons were preincubated with 100 µM of TrkB inhibitor, ANA12 (Sigma-Aldrich, St. Louis, MO), 1 µM of Trk inhibitor, K252a (Sigma-Aldrich, St. Louis, MO) or 1 µM of the FGFR1 inhibitor, PD166285 (Sigma-Aldrich, St. Louis, MO) for 30 min before adding 50 nM recombinant CPE-WT or CPE-E342Q (custom synthesized, GenScript, Piscataway, NJ), or BSA (Sigma-Aldrich, St. Louis, MO) 24h, and then challenged with or without 100µM H_2_O_2_ (Fisher Scientific, Hampton, NH) for 6h_._ Cytotoxicity was evaluated by the LDH release assay.

**Analysis of BCL2 expression in HT22*^cpe-/-^*** **cells treated with H_2_O_2_**

To study the effect of H_2_O_2_ in the presence of recombinant CPE-E342Q on BCL2 expression, HT22*^cpe-/-^* cells were seeded in a 12 well plate. A day later, cells were treated with 100 µM H_2_O_2_ for 24 h. in the presence or absence of 50 nM recombinant WT or E342Q CPE protein. To study the effect of ERK inhibitor on CPE induced BCL2 expression. HT22*^cpe-/-^* cells were treated with 5 μM MEK 1/2 inhibitor U0126 (Sigma-Aldrich, St. Louis, MO) for 30 min. These cells were then treated with 50 nM WT or E342Q recombinant CPE for 6 hours followed by 100 μM H_2_O_2_ treatment for next 24 h. BCL2 expression was assessed by western blot using BCL2 antibody (1:1000 dilution, Cell signaling). β-actin (anti β-actin 1:2000, Cell signaling) was used as a loading control for normalization and these experiments were repeated at least three times.

**Analysis of ERK phosphorylation in HT22*^cpe-/-^* cells**

The effect of WT CPE on ERK 1/2 phosphorylation was analyzed in HT22*^cpe-/-^* cells. Cells (2x10^5^/well) were seeded in 12 well plate in cell culture medium containing 10% FBS and incubated overnight at 37°C in a CO_2_ incubator followed by 3h incubation in serum free medium. These serum starved cells were then treated with different concentrations (0 to 50 nM) of WT-CPE at different time points. Cells were then lysed in Pierce lysis buffer containing Phosphatase inhibitor (Roche, Germany) and analyzed by Western blotting.

Western blot membranes were simultaneously labelled with Phospho ERK1/2 (Thr202/Tyr204) rabbit antibody (1:2500, Cell signaling, Danvers, MA, Cat.#9101 ) and total ERK1/2 mouse monoclonal antibody (1:2500, Cell signaling, Danvers, MA, Cat..#4696 ). Fluorescence labelled anti-rabbit (800 nm, Cat.#926-32213) and anti-mouse (680 nm, Cat.#926-68022) secondary antibodies (1:5000) were used to visualize the protein bands in Odyssey infrared imaging system (Li-COR Inc, Lincoln, NE). Phosphorylated ERK bands were normalized with total ERK bands using Image J software and fold change was calculated. The effect of recombinant CPE E342Q on ERK phosphorylation was also analyzed at different time points using the same procedure.

**Immunohistochemistry and Nissl stain of mouse brains**

The brains were sectioned coronally at 30 µm for Nissl, CPE, doublecortin (DCX) and MAP2 staining. Nissl staining was performed as previously described ^8^. The images were taken with fluorescence microscope (Nikon, Eclipse 80i, Tokyo, Japan) at 4X objective.  Sections were stained with goat anti-CPE antibody (1:3000, R&D systems, Minneapolis, MN, Cat.#3587), polyclonal rabbit doublecortin (DCX) antibody (1:2000, Abcam, Cambridge, MA, Cat.#18723), or guinea  pig anti-MAP2 antibody (1:1000, Synaptic System, Goettingen, Germany, Cat.#188004). For CPE staining, images were taken with fluorescence microscope (Nikon, Eclipse 80i, Tokyo, Japan) at 4X objective. DCX positive cells in the dentate gyrus were counted on the confocal microscope (Zeiss LSM 510 Inverted Meta, Carl Zeiss Microscopy, Thornwood, NY) at 20X magnification and images were taken at 20X and 60X magnification. DCX positive cells were counted in the dentate gyrus (six sections per animal, 3 animals per genotype). The average number of DCX positive cells present in the dentate gyrus were WT=191.66, E342Q=169.66, KO=56.6. For MAP2 staining of hippocampal CA1 region and hilus, images were taken from three random areas (60x60µm) at 20X and three random areas (30x30µm) at 60X for each animal and the intensity of the areas measured. For MAP2 quantification of hypothalamus and prefrontal cortex, three random areas (60x60µm) at 20X in each animal were taken and intensity were measured. The intensity for the total 9 measurements for all 3 animals were averaged.

**Corticosterone levels after weaning stress**

Mice were either kept in the home cage or separated from parents, ear tagged and tail clipped. Cortisone levels were measured 5 min after weaning procedure and analyzed by corticosterone DetectX kit (Arbor Assays,Ann Arbor,Michigan).

**SUPPLEMENTARY FIGURES**

**Figure S1**





**Weaning stress increased corticosterone level in mice.** Corticosterone level was significantly increased in 3 week old WT mice that underwent the weaning stress procedure, but not in mice without weaning. Student t-test, ^+^p<0.0001 for weaning stressed mice compared with non-stressed mice. n=6 mice, values are mean ± SEM.

**Figure S2**


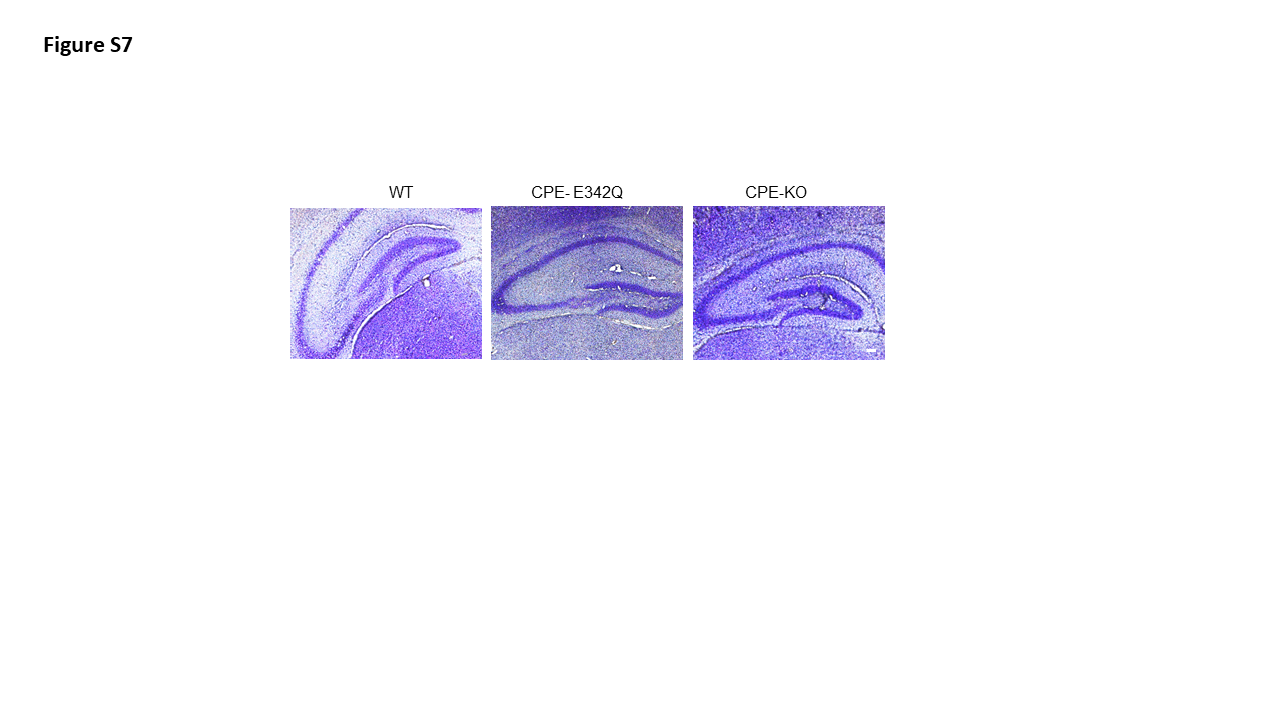


Nissl staining of coronal anterior hippocampus of 3-week old WT, CPE-E342Q and CPE-KO mice. CPE-KO and CPE-E342Q mice display normal hippocampus similar to WT mice at week 3 after toe clipping within postnatal day 7. Scale bar=100 µm.

**Figure S3**


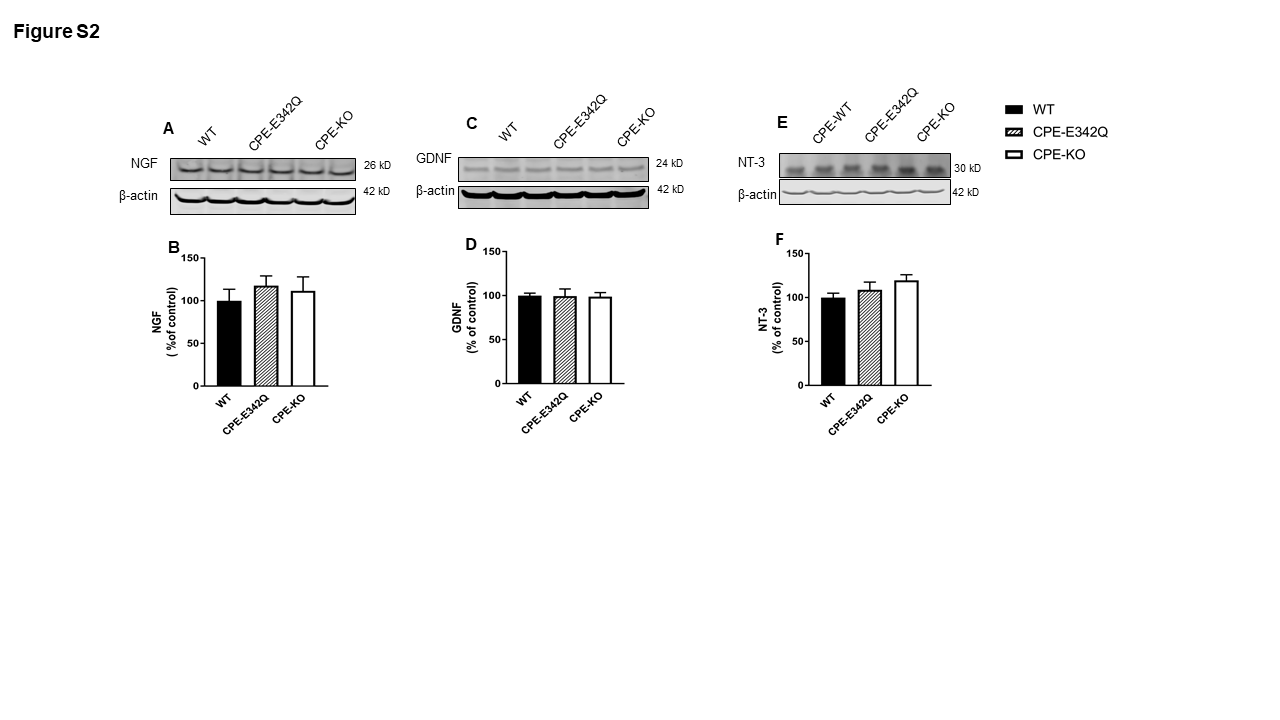


**Expression of neurotrophic factors in the hippocampus of CPE-KO and CPE-E342Q mice is similar to WT mice.**

Since a variety of neurotrophic factors have been known to promote neuroprotection during stress, the protein levels of the most important neurotrophic factors were evaluated in the hippocampus of 12 week old WT, CPE-KO and CPE-E342Q mice.

**(A)** Representative western blot and **(B)** quantification showing NGF protein level in the hippocampus of WT, CPE-E342Q and CPE-KO mice. NGF was not significantly changed in the hippocampus of WT, CPE-E342Q or CPE-KO mice. One-way ANOVA analysis followed by Tukey’s *post-hoc* multiple comparison test, [F_(2,9)_=0.414,p=0.673]. p=0.656 for E342Q mice compared with WT; p=0.831 for CPE-KO mice compared with WT.n=4 mice, values are mean ± SEM.

**(C)** Representative western blot and **(D)** quantification showing GDNF protein level in the hippocampus of WT, CPE-E342Q and CPE-KO mice. GDNF was not significantly changed in the hippocampus of WT, CPE-E342Q or CPE-KO mice. One-way ANOVA analysis followed by Tukey’s *post-hoc* multiple comparison test,[F_(2,9)_=0.011,p=0.989]. p= 0.997 for E342Q mice compared with WT;p=0.988 for CPE-KO mice compared with WT. n=4 mice, values are mean ± SEM.

**(E)** Representative western blot and **(F)** quantification showing NT-3 protein level in the hippocampus of WT, CPE-E342Q and CPE-KO mice. NT-3 was not significantly changed in the hippocampus of WT, CPE-E342Q or CPE-KO mice. One-way ANOVA analysis followed by Tukey’s *post-hoc* multiple comparison test, [F_(2,9)_=2.053,p=0.184].p=0.645 for E342Q mice compared with WT;p=0.162 for CPE-KO mice compared with WT. n=4 mice, values are mean ± SEM.

**Figure S4**

**A. Strategy to generate mouse model carrying the CPE-E342Q mutation**


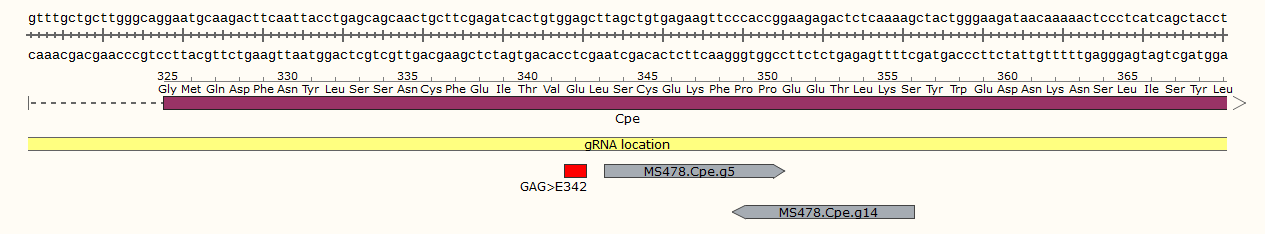


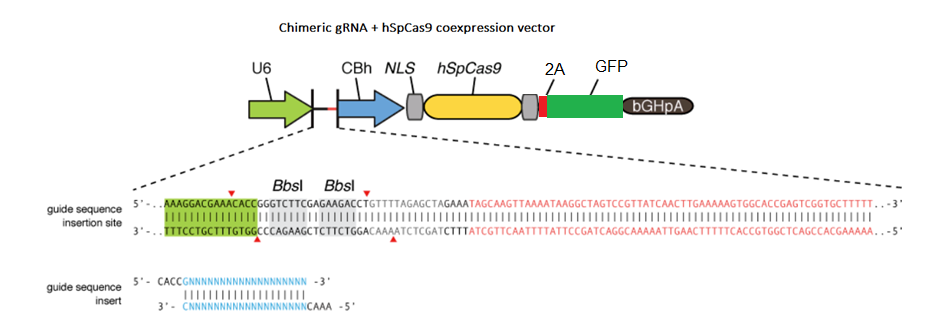


a. 5’GTATGTGTACACTGACTCTCTGTTTGCTGCTTGGGCAGGAATGCAAGACTTCAATTACCTGAGCAGCAACTGCTTCGAGATCACTGTGCAACTTAGCTGTGAGAAGTTCCCACCCGAAGAGACTCTCAAAAGCTACTGGGAAGATAACAAAAACTCCCTCATCAGCTACCTGGAGCAGGT 3’

b.


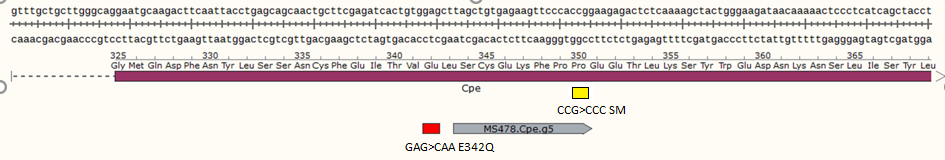


A: Homology Directed Repair (HDR) through embryonic injection.

(a) donor oligo DNA;

(b) editing scheme to create mCpe- E342Q model. Red and yellow letters indicate GAG>CAA (E>Q) mutation and silent mutation CCG>CCC, respectively.

**B**


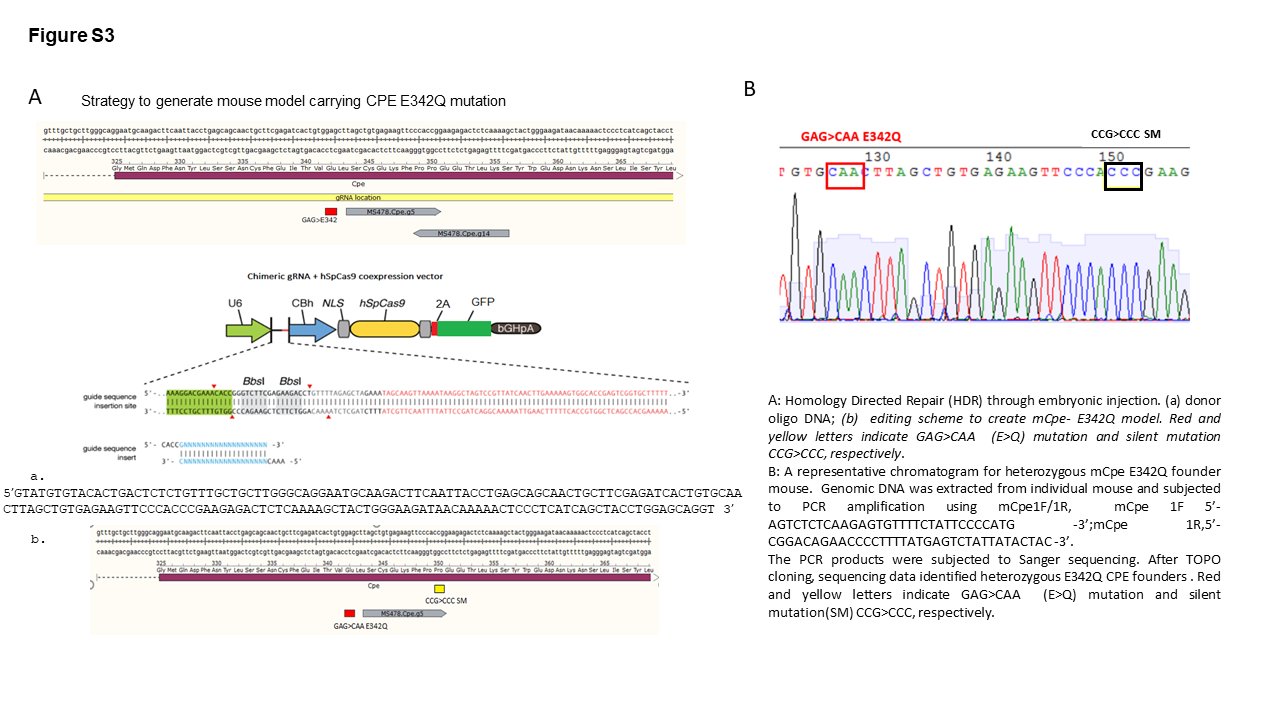


B: A representative chromatogram for heterozygous mCpe E342Q founder mouse. Genomic DNA was extracted from individual mouse and subjected to PCR amplification using mCpe1F/1R, mCpe 1F 5’- AGTCTCTCAAGAGTGTTTTCTATTCCCCATG -3’;mCpe 1R,5’- CGGACAGAACCCCTTTTATGAGTCTATTATACTAC -3’.

The PCR products were subjected to Sanger sequencing. After TOPO cloning, sequencing data identified heterozygous E342Q CPE founders. Red and black boxes indicate GAG>CAA (E>Q) mutation and silent mutation(SM) CCG>CCC, respectively.

**Figure S5**

**
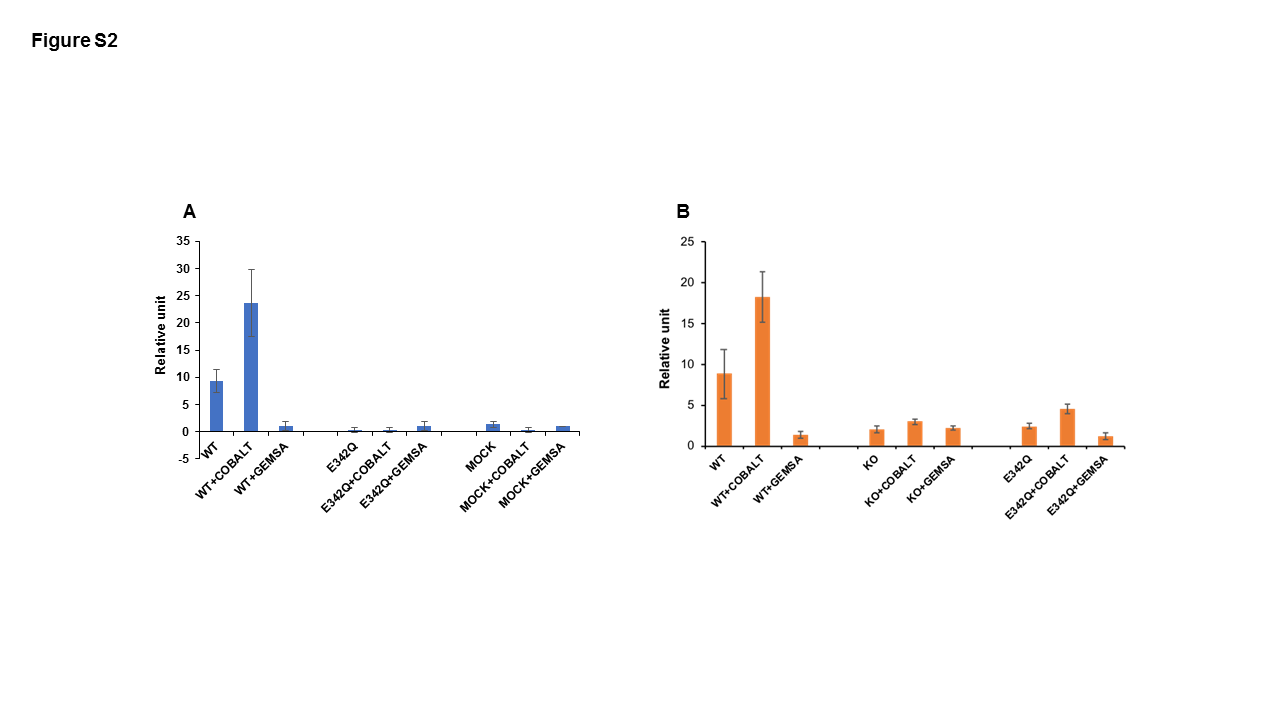
**

**(A)** Recombinant CPE-E342Q does not have enzymatic activity *in vitro*. Enzymatic activity of CPE-E342Q protein was tested by using dansyl-Phe-Ala-Arg substrate. In these experiments 100 ng purified CPE-WT or CPE-E342Q proteins were incubated with 100 μl substrate (0.5mM) for 16 h at 37°C in the presence or absence of cobalt which stimulates and/or GEMSA, which inhibits CPE activity. No enzymatic activity was observed with CPE-E342Q protein in the presence and absence of cobalt. Values shown are mean ± SEM, n=3.

**(B)** CPE-E342Q does not have enzymatic activity *in vivo*. For *in vivo* enzymatic activity assessment, 500 ng total protein extract from pituitary tissue from CPE- WT, KO or E342Q mice were incubated with 100 μl dansyl-Phe-Ala-Arg substrate (0.5mM) for 16 h at 37°C, in the presence or absence of cobalt and/or GEMSA. Bar graphs show CPE-E342Q does not have any enzymatic activity similar to CPE-KO animals. Values shown are mean ± SEM, n=3.

**Figure S6**

**
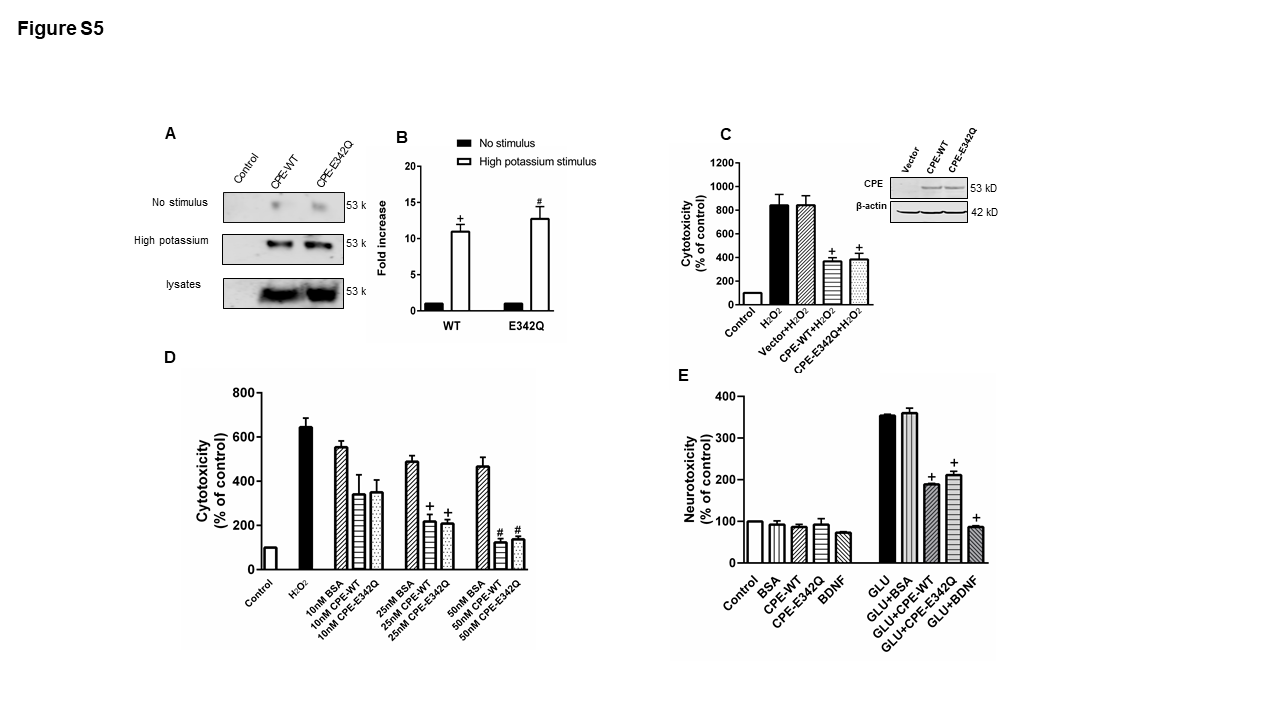
**

**CPE and CPE-E342Q protect HT22*^cpe-/-^* cells from H_2_O_2_ or glutamate-induced neurotoxicity, independent of its enzymatic activity**

**(A)**Representative Western blot of cell lysates, basal medium or stimulated medium collected from HT22*^cpe-/-^* cells transfected with empty vector (control) or vector carrying CPE-WT or CPE-E342Q. **(B)**Bar graphs from quantification of Western blots in (A) showing that secretion of CPE-WT and CPE-E342Q in the HT22*^cpe-/-^* cells was increased with high potassium stimulation. Student *t*-test, ^+^p=0.0006 for WT stimulated compared with WT basal level, ^#^p=0.0023 for E342Q stimulated compared with E342Q basal level. The values are the mean ±SEM, N=3.

**(C)**Transfection of CPE-WT and CPE-E342Q protects HT22*^cpe-/-^* cells against H_2_O_2_-induced cytotoxicity. Bar graphs showing that cytotoxicity was increased significantly after 100µM H_2_O_2_ treatment assessed by LDH assay; however, transfection of CPE-WT and CPE-E342Q reversed this cytotoxicity. One-way ANOVA analysis followed by Tukey’s *post-hoc* multiple comparison test, [F_(4,10)_=28.77, p<0.0001]. ^+^p=0.0018 for WT-CPE+H_2_O_2_, ^+^p=0.0023 for CPE-E342Q+H_2_O_2_, both compared with Vector+H_2_O_2_. The values are the mean ±SEM, N=3, with each experiment done in triplicates.

**(D)** CPE-WT and CPE-E342Q recombinant protein protects HT22*^cpe-/-^* cells against H_2_O_2_-induced cytotoxicity. Bar graphs showing recombinant CPE-WT and CPE-E342Q at 25 and 50nM had neuroprotective effects against H_2_O_2_-induced cytotoxicity as measured by LDH assay. One-way ANOVA analysis followed by Tukey’s *post-hoc* multiple comparison test, [F_(10,22)_ =21.74, p<0.0001]. ^+^p=0.0037 for CPE-WT, and ^+^p=0.0026 for CPE-E342Q compared with 25nM BSA treatment. ^#^p=0.0002 for WT-CPE, and ^#^p=0.0003 for CPE-E342Q compared with 50nM BSA treatment. The values are the mean ±SEM, N=3, with each experiment done in triplicates.

**(E)** CPE-WT and CPE-E342Q protects HT22*^cpe-/-^* cells against glutamate-induced neurotoxicity. Bar graph showing that 50nM CPE-WT, CPE-E342Q and BDNF protected HT22*^cpe-/-^* cells from 40 µM glutamate-induced neurotoxicity. One-way ANOVA analysis followed by Tukey’s *post-hoc* multiple comparison test, [F_(9,20)_=210.8, p<0.0001]. ^+^p<0.0001 for Glu+CPE-WT,Glu+E342Q,and Glu+BDNF, compared with Glu+BSA treatment. The values are the mean ±SEM, N=3, with each experiment done in triplicates. GLU: Glutamate.

**Figure S7**


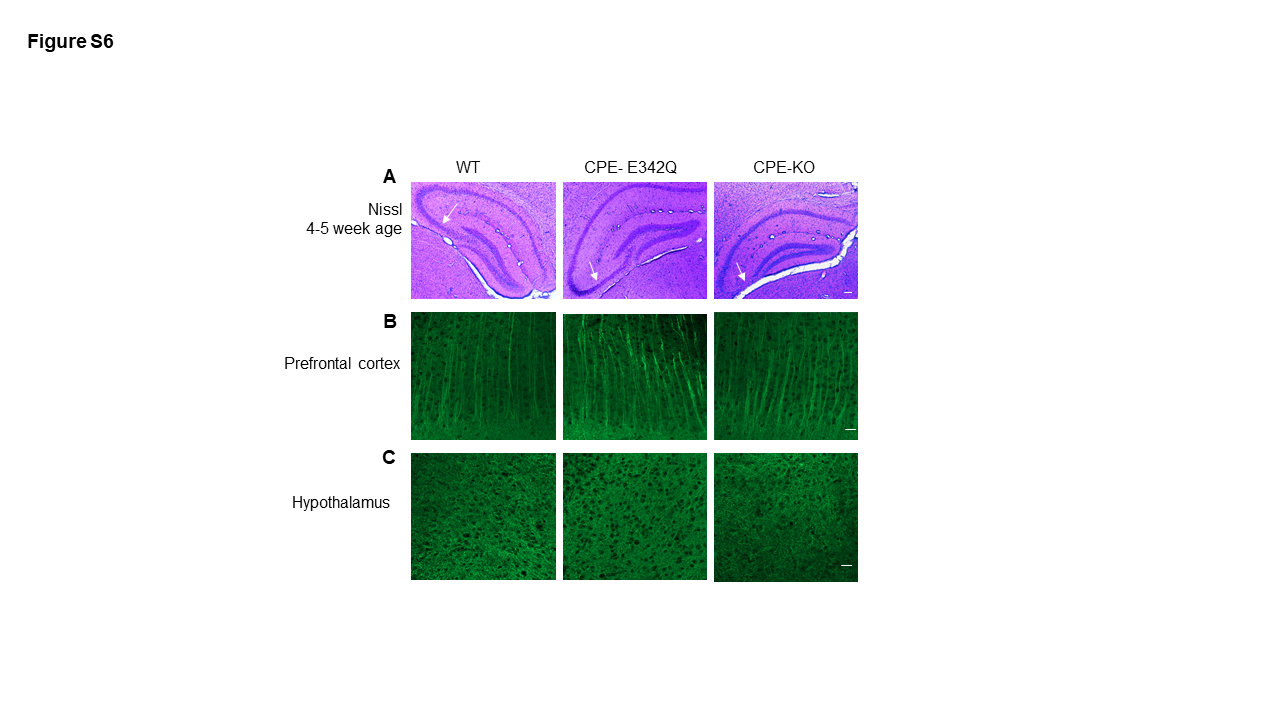


**(A)**Nissl staining of coronal anterior hippocampus of 4-5-week old age of WT, E342Q and CPE-KO mice. CPE-E342Q mice display normal hippocampus similar to WT mice, however, CPE-KO mice demonstrate complete degeneration of CA3 region as arrow indicate. Scale bar=100 µm.

**(B)**MAP2 immunofluorescence staining showing no differences in MAP2 intensity in prefrontal cortex of 12 week old WT, CPE-E342Q and CPE-KO mice. Scale bar=20 µm.

**(C)**MAP2 immunofluorescence staining showing no differences in MAP2 intensity in the hypothalamus of 12 week old WT, CPE-E342Q and CPE-KO mice. Scale bar=20 µm.

**Figure S8**

**
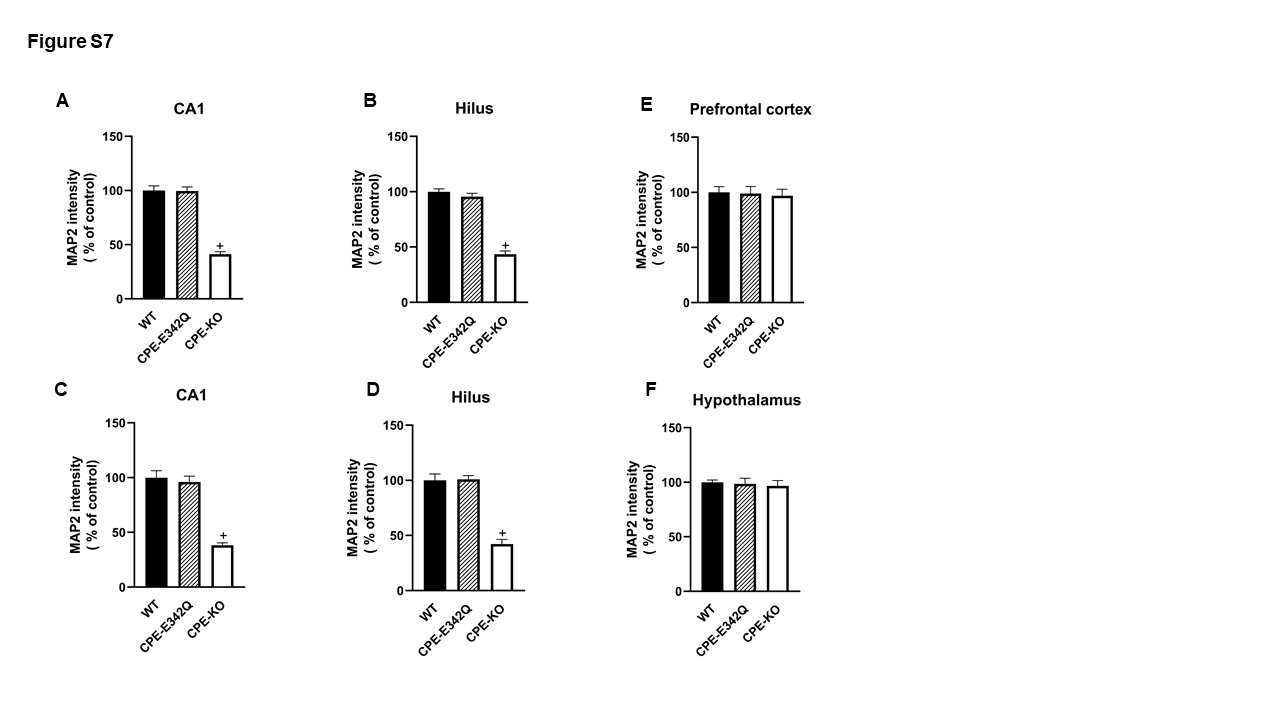
**

**(A)** Bar graphs show quantification of MAP2 immunofluorescence staining intensity of images captured at 20X in hippocampal CA1. There was a significant decrease in MAP2 intensity in the CA1 of CPE-KO, but not CPE-E342Q mice,compared with WT mice. One-way ANOVA analysis followed by Tukey’s *post-hoc* multiple comparison test. [F_(2,24)_=87.61, p<0.0001]. +p<0.0001 for CPE-KO compared with WT; p<0.0001 for CPE-KO compared with CPE-E342Q mice; p=0.996 for CPE-E342Q compared with WT. n=3 per genotype. The values are the mean ± SEM.

**(B)** Bar graphs show quantification of MAP2 immunofluorescence staining intensity of images captured at 20X in hippocampal hilus. There was a significant decrease in MAP2 intensity in the hilus of CPE-KO, but not CPE-E342Q mice, compared with WT mice. One-way ANOVA analysis followed by Tukey’s *post-hoc* multiple comparison test. [F_(2,24)_=119.9, p<0.0001]. +p<0.0001 for CPE-KO compared with WT; p<0.0001 for CPE-KO compared with CPE-E342Q mice;p=0.518 for CPE-E342Q compared with WT. n=3 per genotype. The values are the mean ± SEM.

**(C)**Bar graphs show quantification of MAP2 immunofluorescence staining intensity of images captured at 60X in hippocampal CA1. There was a significant decrease in MAP2 intensity in the CA1 of CPE-KO, but not CPE-E342Q mice, compared with WT mice. One-way ANOVA analysis followed by Tukey’s *post-hoc* multiple comparison test. [F_(2,24)_=49.33, p<0.0001]. +p<0.0001 for CPE-KO compared with WT; p<0.0001 for CPE-KO compared with CPE-E342Q mice; p=0.839 for CPE-E342Q compared with WT. n=3 per genotype. The values are the mean ± SEM.

**(D)**Bar graphs show quantification of MAP2 immunofluorescence staining intensity of images captured at 60X in hippocampal hilus. There was a significant decrease in MAP2 intensity in the hilus of CPE-KO, but not CPE-E342Q mice, compared with WT mice. One-way ANOVA analysis followed by Tukey’s *post-hoc* multiple comparison test. [F_(2,24)_=54.33, p<0.0001]. +p<0.0001 for CPE-KO compared with WT; p<0.0001 for CPE-KO compared with CPE-E342Q mice;p=0.988 for CPE-E342Q compared with WT. n=3 per genotype. The values are the mean ± SEM.

**(E)**Bar graphs show quantification of MAP2 immunofluorescence staining intensity of images captured at 20X in prefrontal cortex of 12 week old WT, CPE-E342Q and CPE-KO mice. There was no significant difference between CPE-KO, WT and CPE-E342Q mice. One-way ANOVA analysis followed by Tukey’s *post-hoc* multiple comparison test, [F_(2,24)_=0.068, p=0.935]. n=3 per genotype. The values are the mean ± SEM.

**(F)** Bar graphs show quantification of MAP2 immunofluorescence staining intensity of images captured at 20X in the hypothalamus of 12 week old WT, CPE-E342Q and CPE-KO mice. There was no significant difference between CPE-KO, WT and CPE-E342Q mice. One-way ANOVA analysis followed by Tukey’s *post-hoc* multiple comparison test, [F_(2,24)_=0.153, p=0.859]. n=3 per genotype. The values are the mean ± SEM.

**Figure S9**

**
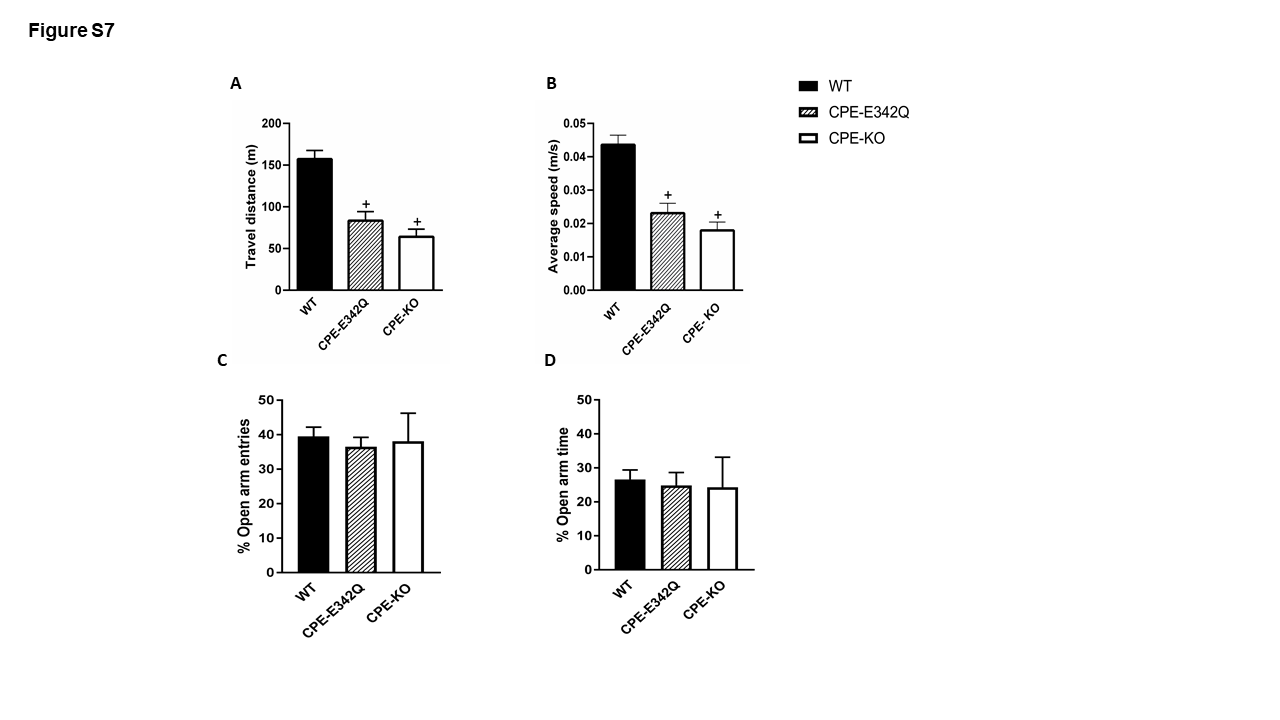
**

**(A)**Travel distance of WT, CPE-KO and CPE-E342Q mice in open field test. Travel distance of CPE-KO and CPE-E342Q were both decreased in comparison with WT. One-way ANOVA analysis followed by Tukey’s *post-hoc* multiple comparison test, [F _(2,45)_ =22.32, p<0.0001]. ^+^p<0.0001 for both CPE-E342Q and CPE-KO mice compared with WT. n=24 for WT, n=12 for CPE-E342Q,n=12 for CPE-KO. The values are the mean ± SEM.

**(B)**Average speed of WT, CPE-KO and CPE-E342Q mice in open field test. Travel speed of CPE-KO and CPE-E342Q were both decreased in comparison with WT. One-way ANOVA analysis followed by Tukey’s *post-hoc* multiple comparison test, [F_(2,45)_=22.23, p<0.0001]. ^+^p<0.0001 for both CPE-E342Q and CPE-KO mice compared with WT. n=24 for WT, n=12 for CPE-E342Q,n=12 for CPE-KO.The values are the mean ± SEM.

**(C)** Percentage of open arm entries in the elevated plus maze. CPE-KO and CPE-E342Q mice displayed similar percentage of open arm entries as with WT mice. One-way ANOVA analysis followed by Tukey’s *post-hoc* multiple comparison test, [F_(2,45)_=0.121,p=0.886].n=24 for WT, n=12 for CPE-E342Q,n=12 for CPE-KO. Values are mean ± SEM.

**(D)** Percentage of open arm time in the elevated plus maze. CPE-KO and CPE-E342Q mice did not show greater percentage of open arm time than WT mice. One-way ANOVA analysis followed by Tukey’s *post-hoc* multiple comparison test, [F_(2,45)_=0.068, p=0.933]. n=24 for WT, n=12 for CPE-E342Q,n=12 for CPE-KO.Values are mean ± SEM.

**References**

1. Davis, J.B. & Maher, P. Protein kinase C activation inhibits glutamate-induced cytotoxicity in a neuronal cell line. *Brain Res* **652**, 169-173 (1994).

2. Lou, H.*, et al.* Carboxypeptidase E cytoplasmic tail mediates localization of synaptic vesicles to the pre-active zone in hypothalamic pre-synaptic terminals. *J Neurochem* **114**, 886-896 (2010).

3. Holmes, A.*, et al.* Galanin GAL-R1 receptor null mutant mice display increased anxiety-like behavior specific to the elevated plus-maze. *Neuropsychopharmacology* **28**, 1031-1044 (2003).

4. Cheng, Y.*, et al.* A human carboxypeptidase E/NF-alpha1 gene mutation in an Alzheimer's disease patient leads to dementia and depression in mice. *Transl Psychiatry* **6**, e973 (2016).

5. Cheng, Y.*, et al.* Neurotrophic factor-alpha1 prevents stress-induced depression through enhancement of neurogenesis and is activated by rosiglitazone. *Mol Psychiatry* **20**, 744-754 (2015).

6. Murthy, S.R.*, et al.* Carboxypeptidase E protects hippocampal neurons during stress in male mice by up-regulating prosurvival BCL2 protein expression. *Endocrinology* **154**, 3284-3293 (2013).

7. Cawley, N.X.*, et al.* Obese carboxypeptidase E knockout mice exhibit multiple defects in peptide hormone processing contributing to low bone mineral density. *Am J Physiol Endocrinol Metab* **299**, E189-197 (2010).

8. Woronowicz, A.*, et al.* Absence of carboxypeptidase E leads to adult hippocampal neuronal degeneration and memory deficits. *Hippocampus* **18**, 1051-1063 (2008).
